# The role of epigenetic modifications, long-range contacts, enhancers and topologically associating domains in the regulation of glioma grade-specific genes

**DOI:** 10.1038/s41598-021-95009-3

**Published:** 2021-08-02

**Authors:** Ilona E. Grabowicz, Bartek Wilczyński, Bożena Kamińska, Adria-Jaume Roura, Bartosz Wojtaś, Michał J. Dąbrowski

**Affiliations:** 1 Institute of Computer Science of the Polish Academy of Sciences, Warsaw, Poland; 2 Faculty of Mathematics, Informatics and Mechanics, University of Warsaw, Warsaw, Poland; 3 Nencki Institute of Experimental Biology of the Polish Academy of Sciences, Warsaw, Poland

**Keywords:** Cancer, Cancer genetics, Cancer genomics, CNS cancer, Paediatric cancer, Computational biology and bioinformatics, Data processing, Databases, Gene ontology, High-throughput screening, Genetics, Cancer genetics, Cancer genomics, Epigenetics, Epigenomics, Gene expression, Gene regulation, Molecular biology, Epigenetics, Transcription

## Abstract

Genome-wide studies have uncovered specific genetic alterations, transcriptomic patterns and epigenetic profiles associated with different glioma types. We have recently created a unique atlas encompassing genome-wide profiles of open chromatin, histone H3K27ac and H3Kme3 modifications, DNA methylation and transcriptomes of 33 glioma samples of different grades. Here, we intersected genome-wide atlas data with topologically associating domains (TADs) and demonstrated that the chromatin organization and epigenetic landscape of enhancers have a strong impact on genes differentially expressed in WHO low grade versus high grade gliomas. We identified TADs enriched in glioma grade-specific genes and/or epigenetic marks. We found the set of transcription factors, including REST, E2F1 and NFKB1, that are most likely to regulate gene expression in multiple TADs, containing specific glioma-related genes. Moreover, many genes associated with the cell–matrix adhesion Gene Ontology group, in particular 14 *PROTOCADHERINs*, were found to be regulated by long-range contacts with enhancers. Presented results demonstrate the existence of epigenetic differences associated with chromatin organization driving differential gene expression in gliomas of different malignancy.

## Introduction

Gliomas are primary brain tumors originating from neural stem cells or progenitor cells^1^. They range from benign and highly-curable pilocytic astrocytomas (World Health Organisation, WHO grade I, GI), through diffuse astrocytomas that could be benign (WHO grade II, GII) or malignant (WHO grade III, GIII) to highly malignant glioblastomas (WHO grade IV, GIV)^2^. Glioblastoma remains an incurable disease with a median survival of 15 months upon treatment and 3 months without treatment^3^. Certain genetic alterations are currently used in glioma classification and survival prognostication, such as 1p/19q co-deletion, *ATRX* and *IDH1/2* mutations^4^. *IDH* mutated gliomas display specific alterations of DNA methylation patterns^5^.

Epigenetic alterations occur in different types of cancers including gliomas^6–10^. For example, DNA methylation of the *MGMT* gene promoter in gliomas leads to gene silencing and is a favourable prognostic marker for patients treated with temozolomide^6^. Methylation of a set of specific cytosines allows for highly accurate prediction of overall survival of glioma patients from TCGA datasets^11^. The recent study of Stępniak et al.^12^ characterized the landscape of open chromatin and histone marks in gliomas of different grades providing a rich resource for further exploration. Gene expression patterns in glioma of different grades have been correlated with epigenetic marks depositions in the transcription start site (TSS) regions. In particular, the higher signals of H3K4me3 have been observed in pilocytic astrocytomas (PA) than in diffuse astrocytomas (DA) and glioblastomas (GBM). In this study we took advantage of that data resource^12^.

Chromatin openness, examined with DNAse I-seq or ATAC-seq assays, correlates positively with transcriptional activity^13,14^. Open chromatin—marked with H3K4me1 but depleted of H3K4me3, has been associated with enhancers^15,16^. Enhancers contain numerous transcription factor (TF) binding sites with their characteristic TF-motifs. TFs can act in either generic or cell-type specific manner^17^. Active enhancers are enriched for H3K27ac while repressed ones for H3K27me3^18,19^.

Another layer of gene regulation complexity is the organisation of the genome into three-dimensional domains, called topologically associating domains (TADs). TAD borders align with H3K27me3 or H3K9me2 blocks, lamin-associated domains as well as coordinately regulated gene clusters^20^. They are stable across different cell types, highly conserved across species^21^ and their disruption can lead to aberrant contacts between genes and enhancers resulting in developmental diseases^22^ and cancer^23,24^. Mutations in the CTCF motif within TAD borders may affect its binding affinity and change expression of sets of genes^25^. *IDH1/2* mutation changing DNA methylation and/or histone methylation may lead to CTCF binding dysfunction in gliomas causing aberrant *PDGFRA* activation^26^.

Here, we took advantage of the wealth of data from the following high-throughput experiments: ATAC-seq, H3K4me3 and H3K27ac ChIP-seqs, DNAse I-seq, DNA bisulfite sequencing and RNA-seq acquired from bulk glioma samples of various WHO grades (GI-GIV). We demonstrated that the epigenetic landscape of promoters and enhancers regulates expression of malignancy-specific genes. We found TADs unexpectedly enriched in glioma grade specific genes and/or epigenetic marks. Moreover, we identified a set of TFs which putatively regulate gene expression in multiple TADs, including known glioma related TFs such as REST, E2F1 and NFKB1. Many genes associated with the cell–cell adhesion Gene Ontology group, including numerous genes of the *PROTOCADHERIN* gene family, were found to be regulated by long-range contacts defined by the previously published Hi-C experiments performed on the human, developing brain^27^. Importantly, we found a large set of *PROTOCADHERIN* coding genes regulated by just one differentially acetylated enhancer.

## Results

### Patterns of epigenetic mark depositions in genes differentially expressed genes between benign vs malignant gliomas

First, we identified differentially expressed genes (DEGs) between gliomas of different WHO grades GI-GIV. We compared the following: (1) pilocytic astrocytomas (PA, WHO GI) vs diffuse astrocytomas (DA, WHO GII/GIII) (PA vs DA), (2) PA vs glioblastoma (GBM, WHO GIV) and pediatric glioblastoma (pGBM, WHO GIV) samples (PA vs GBM/pGBM) and (3) DA vs GBM/pGBM. For each of the three analyses, the following numbers of DEGs were obtained: 2954 for PA vs DA, 4216 for PA vs GBM/pGBM and 117 for DA vs GBM/pGBM (DESeq2, FDR-corrected p < 0.01). Next, DEGs between PA vs DA and DA vs GBM/pGBM samples were identified separately for DA IDH-mutant and DA IDH-wild type samples. It turned out that there was a large overlap between DEGs obtained with the entire set of DA samples and DA samples separated into groups according to the IDH gene mutation status (Supplementary Figs S1A,B). Therefore, IDH status dependent changes were not further considered and we only focused on DEGs from comparisons with the entire DA set of samples. To verify DEGs obtained for DA vs GBM/pGBM comparison in our cohort of patients, we also identified DEGs for DA vs GBM on a larger number of samples deposited in TCGA dataset. In TCGA, a higher number of DEGs was obtained, albeit, when we considered the same number of the top DEGs in TCGA and our cohort (n = 117 genes) the overlap between both datasets was considerable. There were 88 DEGs in common, which is a highly significant result (hypergeometric test, p = 0.0001). This confirms that DEGs found as differentially expressed in our relatively small cohort provide valid information.

**Figure 1:**
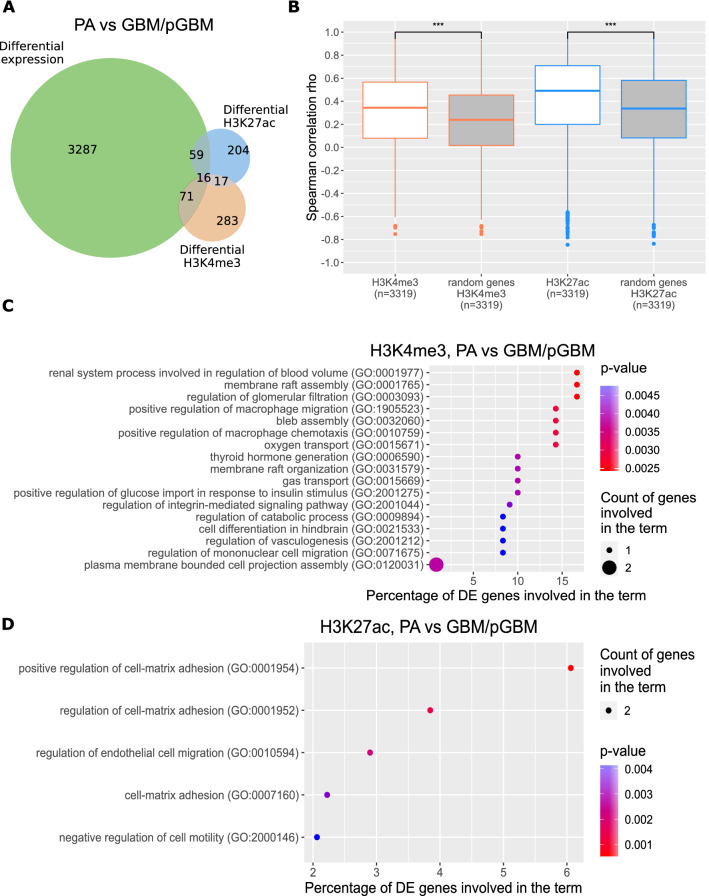
Identification of genes differentially expressed in benign and malignant gliomas (PA vs GBM/pGBM). (**A**) Intersection of differentially expressed genes (DEGs) with genes carrying differential epigenetic modifications (DEMs) for the PA vs GBM/pGBM comparison. (**B**) Correlation of H3K4me3 (orange boxes) and H3K27ac (blue boxes) coverages at the promoters of DEGs (PA vs GBM/pGBM) with their expression. Boxes filled with white show values for DEGs, while in grey for the randomly chosen active genes. (**C**) Enrichment of Biological Process GO terms for DEGs in PA vs GBM/pGBM comparison having high correlation of expression levels with H3K4me3 (Spearman rho > 0.7), and being prognostic for glioma patients’ survival (log-rank test p < 0.001). (**D**) As in (**C**) for H3K27ac.

Some of the identified DEGs had also significantly differential epigenetic marks (DEMs) deposited at the promoter regions (DESeq2, FDR-corrected p < 0.01). The majority of DEGs having deposited DEMs at the promoters were discovered for PA vs GBM/pGBM comparison: there were 75 DEGs with H3K27ac DEMs and 87 with H3K4me3 DEMs (Fig. 1A). When compared to randomly selected, active genes, a significant intersection of DEGs with DEMs suggested that epigenetic regulation plays an important role among genes involved in gliomagenesis (p < 0.01, bootstrapping procedure). For DEGs identified in PA vs DA comparison we found similar results (Supplementary Fig. S1C), in contrast to DA vs GBM/pGBM, where only few DEGs and genes with DEMs were found (Supplementary Fig. S1D).

For further comparison of three glioma grades, DEGs and genes with assigned DEMs were selected. We discovered a significantly stronger correlation between the expression of DEGs and H3K4me3 and H3K27ac deposition at their promoters (0.31–0.42 and 0.57–0.67 for H3K4me3 and H3K27ac, respectively) than in the promoters of randomly selected non-DEG, active genes (Fig. 1B; Supplementary Figs S1E,F). The differences between correlations of DEGs/DEMs and correlations of randomly selected active genes and their epigenetic marks reached statistical significance for all three pairwise comparisons (Wilcoxon test, p < 9e^−187^). These findings suggest that epigenetic marks deposited in the gene promoters might have a significant impact on transcription of genes involved in the development of glioma.

We obtained sets of highly correlating DEGs (Spearman rho > 0.7) with histone modifications: 882, 41, 895 for H3K27ac and 491, 19, 383 for H3K4me3 for three pairwise comparisons: PA vs DA, DA vs GBM/pGBM and PA vs GBM/pGBM, respectively. The sets of DEGs highly correlated with epigenetic marks shared on average 1.28% genes with the set of genes prognostic for patients’ survival (p < 0.001 log-rank test, proteinatlas.org; Supplementary Table S1), while all DEGs shared on average 0.34% genes. The observed difference reached statistical significance (Wilcoxon test p < 0.05). This finding suggests that differences in epigenetic mark levels between tumours of different malignancy grades can affect the expression of key cancer genes, potentially affecting glioma patients’ survival. Functional enrichments of those genes are connected to cell migration and extracellular matrix organization, among other pathways (Figs. 1C,D; Supplementary Fig. S1G–J). When looking more closely into the genes behind GO terms on the Fig. 1D, we see that three genes appear in these terms: *EMP2*, *THY1* and *STC1*. According to GO terms, these genes positively regulate cell–cell adhesion and negatively regulate cell motility (Fig. 1D). We also looked into their prognostic value, and found that all three are prognostic in GBM tumours, with low expression corresponding to a higher overall survival rate (data not shown).

### Organization of chromatin into TADs contributes to expression of genes involved in gliomagenesis

All high-throughput experiments, including ATAC-seq, H3K4me3, H3K27ac ChIP-seqs, DNAse I-seq, DNA bisulfite sequencing, and RNA-seq, revealed that the distribution of signal fold changes between gliomas of different grades followed the structure of TADs organization. The fold changes of signals from all of those experiments computed for all three grade-differential-analyses were found to be more homogeneous within TADs than between TADs (Supplementary Tables S2–S4). The pattern of grade specific changes in chromatin activity and chromatin openness in gliomas is aligned with chromatin segmentation into TADs, according to this finding.

**Figure 2:**
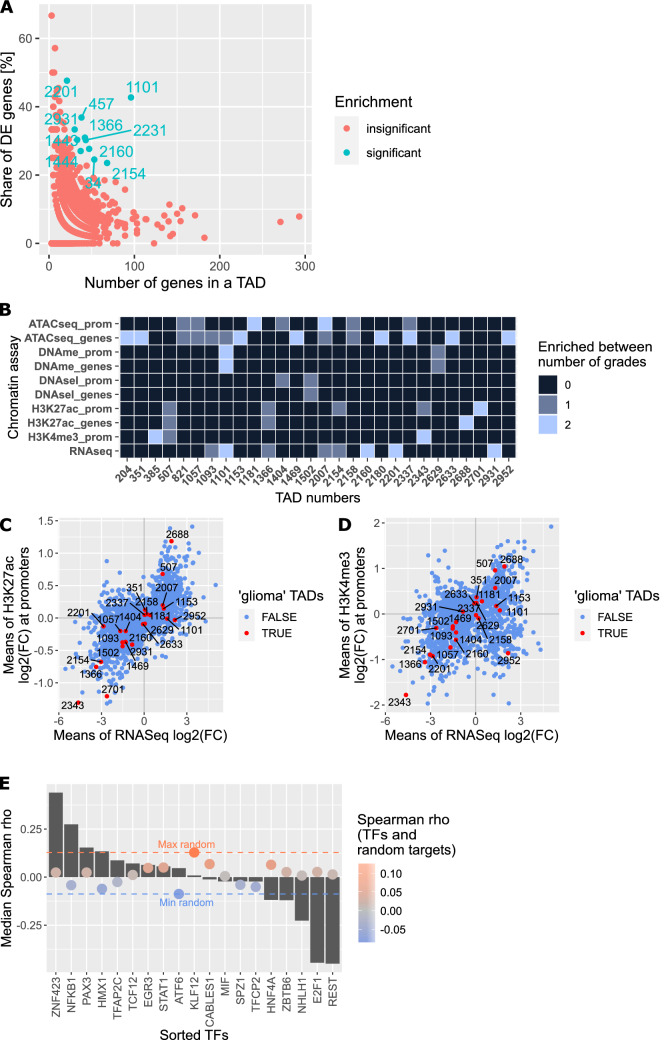
Discovering TADs enriched in genes differentially expressed and epigenetically modified in gliomas of different malignancies. (**A**) TADs enriched for DEGs or genes with DEMs deposited at their promoters, in the PA vs GBM/pGBM comparison. (**B**) TADs with exceptionally high proportion of DEGs and genes carrying DEMs in all grades comparisons. Colour scale depicts in how many of the three grade-comparisons a particular TAD was found to be enriched. (**C**) Dotplot showing means of DEGs expression and H3K27ac peak signals fold changes between PA and GBM/pGBM samples. Each dot represents a mean for each TAD. Dots in red mark the most enriched TADs (‘glioma TADs’, binomial test Benjamini–Hochberg corrected p < 0.05). (**D**) As in Fig. 2C for H3K4me3. (**E**) Spearman correlation between gene expression levels of enriched TFs and their target genes within TAD 2337. Grey bars depict median Spearman correlation between expression levels of genes encoding TFs and their target genes. Red and blue lines demarcate maximal and minimal correlation between expression levels of TF-coding genes and randomly selected, active genes. Colours of dots show median level of Spearman correlation between TFs and random target genes (red—positive correlation, blue—negative correlation).

Furthermore, we aimed to find TADs in gliomas that are particularly rich in DEGs and/or genes with DEMs. We discovered a subset of TADs that were significantly enriched in genes associated with glioma tumorigenesis (binomial distribution test, Benjamini–Hochberg corrected p < 0.05), which we will refer to as ‘glioma TADs’. Those TADs were enriched for DEGs and genes with DEMs including H3K4me3, H3K27ac, DNA methylation, ATAC-seq, DNAse I-seq (Fig. 2A). TAD number 1101, located at chr5: 140 660 415-141 580 433, was by far the most enriched (Fig. 2B; Supplementary Fig. S2A,B).

Besides, we observed that the fold changes of levels of H3K4me3 and H3K27ac deposition (measured as peaks’ heights) between different glioma grades were strongly correlated with fold changes of DEGs expression at the TAD level (Figs. 2C,D, see Supplementary Information for details). The PA vs DA comparison yielded similar results (Supplementary Fig. S2C,D). This suggests that the TAD structure orchestrates gene expression changes in gliomas of various grades, which are accompanied by simultaneous changes in H3K4me3 and H3K27ac epigenetic marks.

The genes found in the ‘glioma TADs’ play significant roles in cancer development (Supplementary Table S5; Supplementary Fig. S3A–D). Genes within the most enriched TAD 1101 encode cadherins which are involved in the cell adhesion process and Wnt signaling pathway. The boundaries of all TADs and genes that reside within them are listed in Supplementary Table S6.

We used RNA-seq data to perform a CNA analysis to account for the potential effect of Copy Number Alterations (CNAs) on gene expression. TAD 1366 was found to be amplified in 4 out of 12 GBM samples analysed (focal chr7 CNA) and 2 of the 6 DA samples (focal chr7 CNA; Supplementary Table S7). There were no amplified regions in the remaining TADs.

We also looked into whether the common transcription factors (TFs) could control the expression of DEGs found in the top six ‘glioma TADs' (Supplementary Table S5). As a result, we were able to generate a list of 75 distinct TFs with strong and significant correlations to their target genes (one example is shown for TAD 2337, Fig. 2E; Supplementary Fig. S4A–C; Supplementary Table S8). The TFs with highest absolute values of correlation were *ZNF423* (rho = 0.44), *REST* (rho = − 0.45), *E2F1* (rho = − 0.45) and *NHLH1* (rho = − 0.23). We identified a group of TFs which putatively regulate gene expression in multiple TADs: E2F1, REST, PAX5, NFE2L1, NFKB1, HMX1 and ZBTB6 (Supplementary Table S5). TFs, known to induce gene expression, were found to be positively correlated with their target genes, while repressors were found to be negatively correlated. In addition, an independent analysis using the BMO tool^28^ confirmed the TF predictions. We detected TF binding sites for E2F1, HMX1, PAX5, REST and ZBTB6 in the promoters of DEGs present within the top six ‘glioma TADs’ (Supplementary Table S9).

### Glioblastoma-related TADs are enriched in DEGs with bivalent chromatin

Glioblastomas are described as frequently having marks of bivalent chromatin within gene promoters^29^. Due to sample availability limitations, we obtained good quality H3K27me3 data only for GBM/pGBM samples and because of that bivalent chromatin detection was performed just for the GBM/pGBM samples. We uncovered 54 DEGs from PA vs GBM/pGBM (Supplementary Table S10) with bivalent chromatin signals in GBM/pGBM samples, which were simultaneously marked by H3K27me3 and H3K4me3. DEGs with bivalent chromatin are associated with positive regulation of cell cycle, cell proliferation, cell adhesion and MAP kinase activity, among others (Supplementary Fig S4D). Six of those genes were found in three of the six most enriched ‘glioma TADs’: 2160 (*CDK4*, *TSFM*, *TSPAN31*, *B4GALNT1*), 2337 (*NFATC4*) and 2688 (*CACNA1G*). This indicates that the TADs rich in genes involved in gliomagenesis are also rich in genes with bivalent chromatin (p < 0.001, hypergeometric test).

### Regulation of DEGs transcription by long-range contacts with enhancers

#### Genes in contact with enhancers

We investigated whether DEGs in contact with enhancers in gliomas have elevated expression levels. Out of DEGs from PA vs GBM/pGBM comparison, 41% (n = 1716) had predicted long-range contacts with at least one enhancer. In our samples, those enhancers accounted for 8.62% (n = 3928) of all predicted enhancers. Some enhancers, on the other hand, had no predicted contacts and others had contacts with other loci than DEGs. DEGs with predicted enhancer contacts had significantly higher expression (by 11%) than DEGs that did not have such contacts (Wilcoxon test, p = 3.5e^−27^).

#### Genes with multiple chromatin contacts

**Figure 3:**
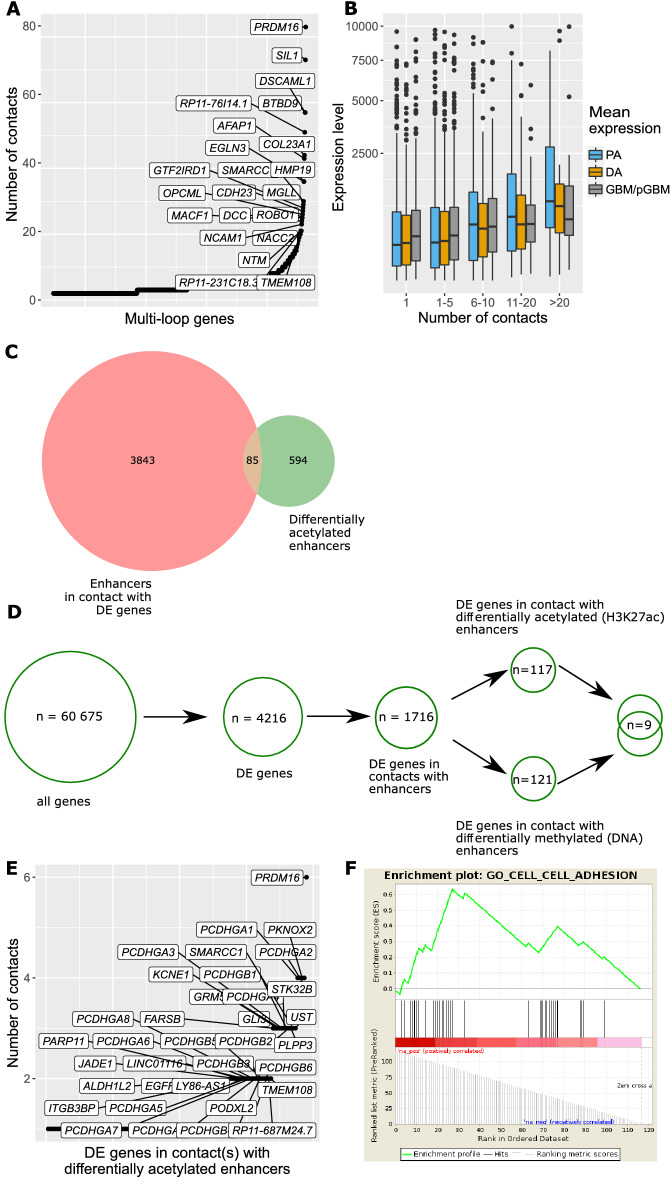
Prediction of gene regulatory networks enriched for multiple predicted contacts with enhancers. (**A**) DEGs from PA vs GBM/pGBM comparison (n = 1041) with predicted multiple contacts with enhancers. (**B**) Higher numbers of contacts with enhancers co-exist with higher expression of DEGs (PA vs GBM/pGBM). (**C**) Enhancers contacting DEGs (PA vs GBM/pGBM) intersect with the differentially acetylated (H3K27ac) (PA vs GBM/pGBM) enhancers. (**D**) Workflow for obtaining DEGs potentially regulated by the differential activity of their contacting enhancers. The obtained enhancer activity might be due to differential H3K27ac and/or DNA methylation marks. Numbers are given for the comparison of PA vs GBM/pGBM. (**E**) DEGs (PA vs GBM/pGBM) contacting the differentially active enhancers (differential H3K27ac marks between PA vs GBM/pGBM). (**F**) The most enriched gene set from GSEA performed on DEGs having at least one contact with differentially acetylated enhancers between PA vs GBM/pGBM glioma samples.

Recent findings showed that genes with multiple loops have usually higher expression than genes with single loops^24,30^. Therefore, we identified genes having multiple contacts with enhancers localized on the same chromosomes (Fig. 3A; Supplementary Fig. S5A, B). Out of the DEGs from PA vs GBM/pGBM comparison, 25% (n = 1041) had predicted contacts with multiple enhancers (in PA vs DA 24% and in DA vs GBM/pGBM 29%, respectively). Gene set enrichment analysis (GSEA) of those genes showed cell–cell adhesion via plasma membrane adhesion molecules (normalized enrichment score (NES): 1.9, q = 0.051) and stress-activated protein kinase signalling cascade (NES: 1.76, q = 0.065) as the most enriched sets. *PRDM16* was the DEG with the most loops, with 83 contacts and 24 enhancers (Fig. 3A). Furthermore, we found that one locus is particularly rich in long-range contacts with regions of unknown function, possibly a regulatory activity. The following DEGs: *SAPCD1-AS1*, *MSH5*, *MSH5-SAPCD1*, *SAPCD1*, *LY6G6C*, *LY6G6D*, and *ABHD16A* were all found in this region. Finally, we confirmed that DEGs with multiple (≥ 6) contacts with enhancers had higher expression in glioma bulk tumour samples than DEGs with only few contacts (1–5), (Wilcoxon test p = 1.1e^−17^, 2.1e^−13^, 0.008 for PA vs GBM/pGBM, PA vs DA and DA vs GBM/pGBM, respectively, Fig. 3B; Supplementary Fig. S5C,D).

To account for possible effect of CNAs on gene expression, we checked whether genes with multiple contacts with enhancers held CNAs. For *PRDM16* focal CNAs were detected in 2 out of 12 GBM samples, while the region around the *MSH5* gene was amplified in 3 out of 6 DA samples, in 7 out of 12 GBM samples, but also in 4 out of 11 PA samples (among the PA samples there was also one deletion). The presence of CNAs is thus unlikely to be the cause of differences in gene expression between PA and GBM/pGBM samples (Supplementary Fig. S5E, Supplementary Table S11).

#### Enhancers with grade-specific activity

Following the observation that not all of the enhancers are active in all patients but they are rather patient-specific^12^, we looked for enhancers that were differentially active in glioma of various grades (Fig. 3C; Supplementary Fig. S5F,G). We found 679 enhancers with significantly different levels of H3K27 acetylation between PA vs GBM/pGBM samples (Wilcoxon test p < 0.01). Out of them, 85 (12.8% of the enhancers with contacts, Fig. 3C) were in contact with 117 DEGs (PA vs GBM/pGBM) (Fig. 3D). *PRDM16* had the highest number of contacts (n = 6) with the differentially acetylated enhancer (chr1: 3078107–3079482) (Fig. 3E). The *PRDM16* gene codes for a histone H3K9 monomethyltransferase. Besides that, a larger number of *PCDHGA* genes were found to be in contact with a differentially acetylated enhancer (chr5: 141528260–141529747) (Fig. 3E). GSEA analysis of 117 DEGs with at least one contact with differentially acetylated enhancers between PA and GBM/pGBM revealed a significant enrichment of genes related to cell–cell adhesion (NES: 1.94, q = 0, Fig. 3F).

#### Grade-specific enhancers may activate PROTOCADHERIN genes

**Figure 4:**
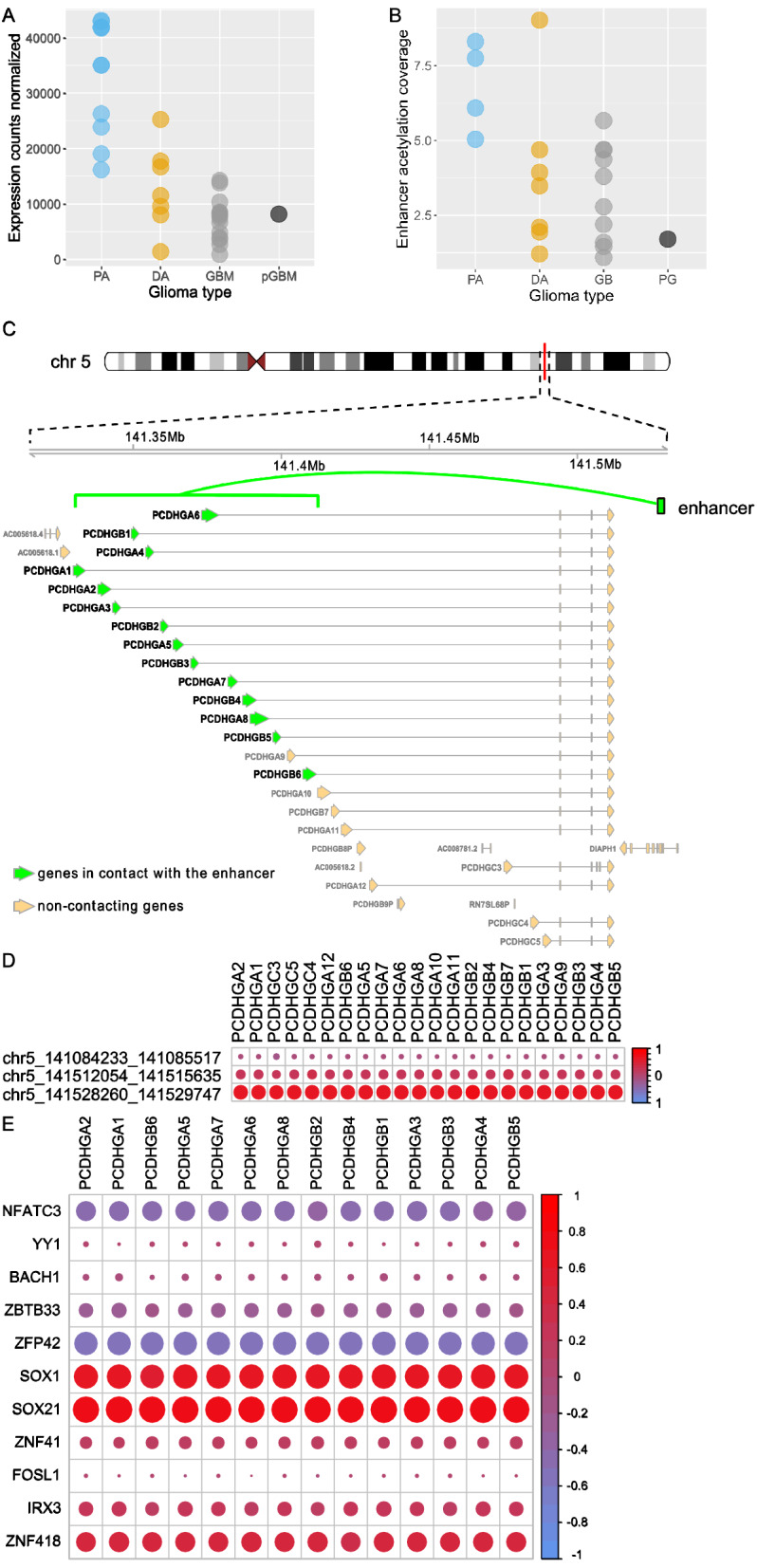
Regulation of *PCDHGA* gene cluster by H3K27 acetylation and/or DNA methylation of their contacting enhancers, as well as possible binding of transcription factors. (**A**) Expression of the *PCDHGA2* gene (as an example of *PCDHGA* genes which had similar expression levels) across glioma grades. (**B**) H3K27 acetylation levels at the enhancer (chr5: 141528260–141529747) contacting *PCDHGA2* gene across glioma grades. (**C**) Visualization of contacts between *PCDHGA* genes cluster and contacting enhancer at chr5: 141528260–141529747. (**D**) Correlations between expression levels of *PCDHGA* genes and H3K27ac levels of their contacting enhancers. (**E**) Correlations between expression levels of *PCDHGA* genes and TF-coding genes which binding sites were detected within the chr5: 141528260–141529747 enhancer. These TFs were identified using DAVID and BMO.

Within the cell–cell adhesion set of genes there were found 14 *PCDHGA* genes that encode protocadherins—calcium-dependent cell-adhesion proteins. One of the ‘glioma TADs’—number 1101—contains a cluster of those genes (Supplementary Table S5). *PCDHGA* genes showed overall high transcription in PA samples, lower in DA and lowest in GBM/pGBM samples (Wilcoxon test p < 0.0005, Fig. 4A). The relatively low expression of those *PCDHGA* genes in pGBM, as in GBM, suggests that their possible dysregulation is linked to cancer malignancy rather than age. PA samples had significantly higher levels of H3K27 acetylation of the differentially acetylated enhancer contacting those genes than DA or GBM/pGBM samples (Wilcoxon test, p = 0.07 PA vs DA, p = 0.003 PA vs GBM/pGBM, Fig. 4B). Of note, only one of the three enhancers contacting the *PCDHGA* genes cluster was differentially acetylated (Fig. 4C). The median correlation between expression of *PCDHGA* genes with the acetylation of the enhancer (chr5: 141528260–141529747) across the samples was high (Spearman rho = 0.73, p = 0.0002, Fig. 4D). Using the BMO tool, we discovered the presence of TF binding sites for multiple TFs within this enhancer (Fig. 4E). It is worth noting that the highest mean positive correlation of expression of *PCDHGA* genes with the genes coding those TFs was observed for *SOX21* and *SOX1* (Spearman rho = 0.72 and 0.62, respectively, Fig. 4E) while the lowest mean correlation was found for *ZFP32* and *NFATC3* (Spearman rho = − 0.57 and − 0.42, respectively). We postulate that binding of those TFs within the chr5: 141528260–141529747 enhancer may influence expression of the *PCDHGA* gene cluster.

#### Enhancer-driven regulation deteriorates with tumour malignancy

**Figure 5:**
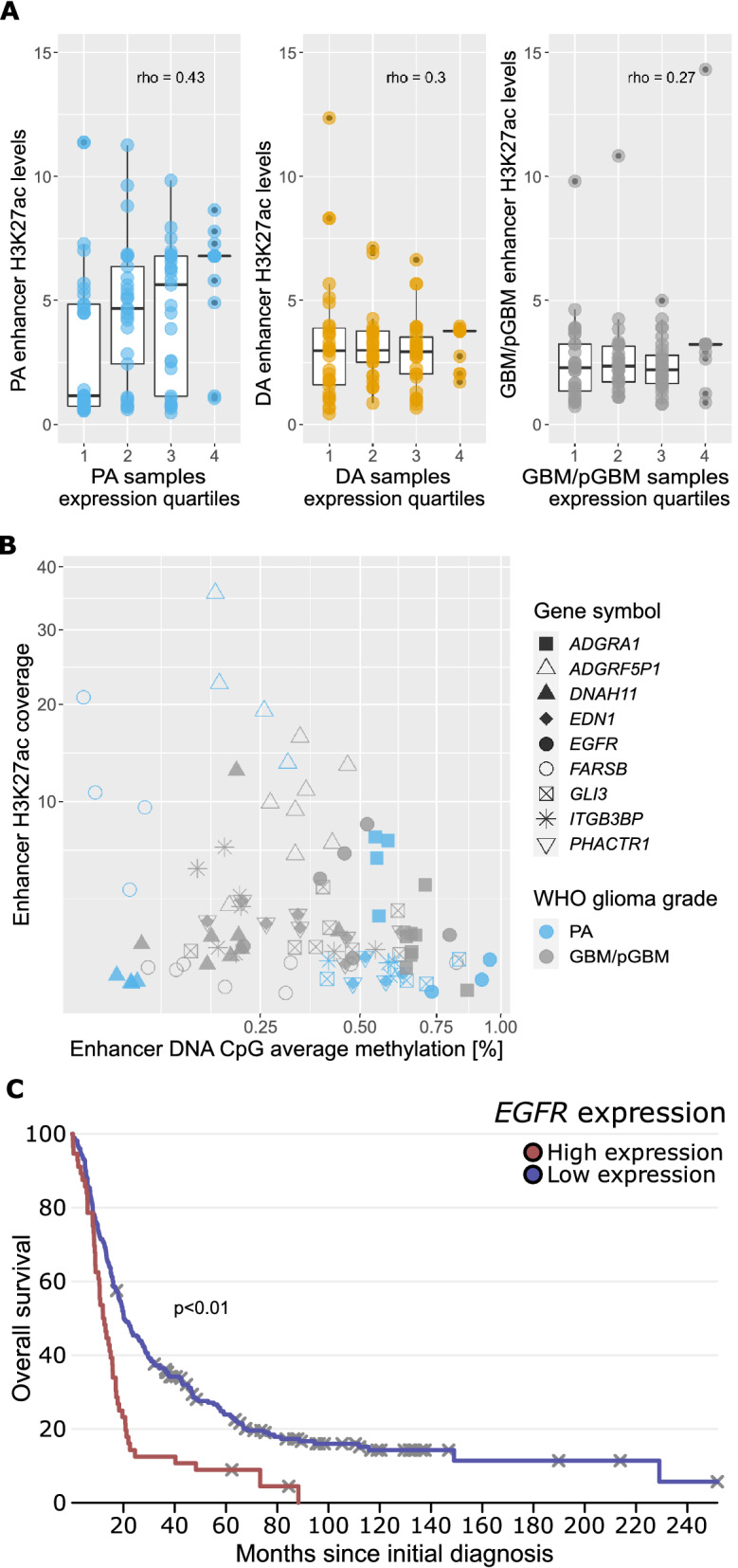
The effect of differential H3K27 acetylation and/or DNA methylation at enhancers on the expression of their target genes: *PROTOCADHERINS*, *EGFR* and other glioma-related genes. (**A**) Correlation between enhancers’ H3K27 acetylation and expression of DEGs (PA vs GBM/pGBM) contacting them. Correlation decreases in higher grade gliomas. (**B**) Correlation between DNA methylation and H3K27ac signals at the enhancers being in contact with DEGs and being differentially methylated and acetylated (H3K27ac) between PA vs GBM/pGBM samples. Each dot represents a gene in each sample—9 genes in 12 samples for which there were simultaneously available good quality DNA methylation and H3K27ac data. (**C**) Survival analysis plot of the expression of *EGFR* gene showing its prognostic value for glioma patients’ survival, based on the Rembrandt dataset.

Strong correlation between enhancer acetylation (H3K27ac) and target gene expression in all 117 genes that contact differentially acetylated enhancers was detected (Supplementary Table S12). The strongest correlation was found in PA samples (Fig. 5A, Supplementary Table S12). When permutation test was applied, the median correlation values were nearly zero (Supplementary Table S12). Moreover, we found that acetylation of DEG-contacting enhancers was higher in PA samples than in higher grade gliomas (DA, GBM/pGBM) (Fig. 5A). Enhancer acetylation was associated with the expression of highly expressed genes in higher grade gliomas, but it was well pronounced across all RNA expression quantiles in PA, increasing towards the upper quantile (Fig. 5A). It shows that high enhancer acetylation in DA and GBM/pGBM samples can boost the expression of genes that are already highly expressed. Overall, this finding suggests that a potential dysregulation of the gene-enhancer network could drive the expression of glioma-related genes.

#### Activity of enhancers might be blocked by DNA methylation

Finally, for the DEGs having contacts with differentially acetylated enhancers (H3K27ac), we determined the role of DNA methylation of the contacting enhancers on gene expression. We found nine differentially acetylated enhancers with differentially methylated DNA (Wilcoxon test, p < 0.01, Fig. 3D) out of 85 differentially acetylated enhancers. The selected nine enhancers had predicted contacts with nine DEGs. For all those genes except the *DNAH11*, negative correlation between DNA methylation and H3K27 acetylation levels of enhancers was detected (median of Spearman rho calculated for all genes separately = − 0.57, median p = 0.05, Fig. 5B, for *DNAH11* Spearman rho = 0.79, p = 0.03, Supplementary Table S13). The enriched GO Biological Process terms for the nine genes were: heart and tongue development, cell surface receptor signalling pathway, dorsal/ventral pattern formation, positive regulation of nitric oxide biosynthetic process, positive regulation of MAP kinase activity and wound healing (David tool, p < 0.05). One of the nine DEGs with predicted contacts with enhancers was *EGFR*, a well-known cancer-related gene whose high expression predicts poorer survival in a variety of cancers, including glioblastomas (Fig. 5C). In our study, *EGFR* was overexpressed in DA and GBM/pGBM samples when compared to PA. At the same time, the enhancer (chr7: 54881593–54882643) related to the *EGFR* gene had significantly higher acetylation and lower DNA methylation in DA and GBM/pGBM samples than in PA (p < 0.01, Wilcoxon test; Spearman correlation of enhancer DNA methylation and H3K27 acetylation rho = − 0.5, p = 0.17, n = 12 samples, , Supplementary Fig. S5H,I). According to TCGA data, half of the genes contacting differentially acetylated and differentially DNA-methylated enhancers, namely *GLI3*, *ITGB3BP*, *DNAH11*, *ADGRA1* and *ADGRF5P1* were found to be prognostic for patients’ survival (log-rank test p < 0.0001, in the TCGA dataset for WHO GIII and GIV glioma patients; Supplementary Table S13, Supplementary Fig. S6A–E). The higher gene expression of *GLI3*, *ITGB3BP*, and *DNAH11* was linked to shorter survival, while *ADGRA1* and *ADGRF5P1* higher gene expression was linked to longer survival. We excluded single nucleotide polymorphisms (SNPs) as a cause of varying levels of enhancer activation because none of the SNPs in tumour DNA were associated with malignancy and had a low population frequency (results not shown).

## Discussion

We studied the epigenetic mechanisms which might contribute to the regulation of expression of genes involved in gliomagenesis. Our findings revealed that an interplay of chromatin organization and different epigenetic mechanisms provides a fine-tuned framework for regulation of gene expression which might lead to and/or sustain gliomagenesis.

Long-distance interactions between genes and enhancers add another layer of regulation. Genes forming multiple contact loops with enhancers have been found to be more highly expressed in glioma stem cells than those not in contacts^27^. We identified a specific region on chromosome 6 (chr6: ~ 30 515 000 ~ 33 450 000) that was abundant in long-range contacts. This region did not have H3K27ac marks in our data and therefore it could not be assigned as an active enhancer, however its high connectivity suggests its putative regulatory function. Interestingly, it is a gene-rich region containing genes which were predicted to be the most frequently contacted (40–97 predicted contacts) with other chromatin regions. The most frequently contacted genes were found within the Major Histocompatibility Complex (MHC) class III gene cluster, which is still poorly understood. MHC class III genes, involved in immunity, cell-signalling, genes coding for complement proteins, cytokines and heat shock proteins lie within the region^31–33^. *MSH5*, one of the most frequently contacted genes, encodes a MutS homolog, which is involved in homologous chromosome recombination and has been linked to DNA damage response and repair, neoplasia and immunity^34,35^. High expression of *MSH5* is associated with shorter survival of glioma patients (log rank test p = 1.08e^−7^, Rembrandt repository)^36^, which is in agreement with our results. We found a higher *MSH5* expression in gliomas of WHO grades II/III and IV.

Additionally, we discovered enhancers with H3K27ac signals that were significantly different in glioma of various grades, indicating that their activity changed as the malignancy grade progressed. The correlation between enhancer H3K27 acetylation and the expression levels of their target genes ranged from 0.27 to 0.43 in gliomas, which was higher than the correlation between all active, non-DE genes in gliomas^12^. Stępniak and coworkers^12^ have analysed the same dataset and the correlations of expression of all active genes with H3K27ac of their contacting enhancers were rather low: 0.1–0.25. Altogether, these findings suggest that a subset of genes identified in this study is strongly regulated by active enhancers and may play a role in gliomagenesis.

The gene contacting the largest number of differentially acetylated enhancers (n = 6) was *PRDM16,* encoding for a zinc finger methyltransferase which monomethylates histone H3K9^37^ and also binds DNA acting as a transcriptional regulator. *PRDM16* was over-expressed in leukemia samples^38^ and is an important regulator of differentiation of myoblastic precursors into brown adipose cells^39^. It can also act as a regulator of Transforming Growth Factor (TGF) β1 expression. *TGFβ1* is overexpressed in gliomas and plays important roles in glioma proliferation, invasion and immunosuppression^40^. Differential H3K27 acetylation of the enhancers contacting the *PRDM16* gene across grades could thus be a factor boosting *PRDM16* expression and possibly contributing to gliomagenesis.

DNA methylation at CpG sites usually silences transcription and has a negative correlation with histone marks enrichment at enhancers^41^. We found glioma-grade specific enhancers-target gene pairs, in which enhancers activity could be impaired by DNA methylation, resulting in weaker transcription activation of their target genes. At enhancers contacting DEGs, we discovered an inverse relationship between CpG methylation and H3K27 acetylation. Interestingly, we found the *EGFR* gene among the genes contacting differentially acetylated (H3K27ac) and differentially methylated (DNA) enhancers. It encodes an epidermal growth factor receptor that regulates cell proliferation and has been linked to cancerogenesis^42–44^. Its high expression is associated with poor prognosis in glioma patients. *EGFR* expression was found to be upregulated in our dataset and to be elevated in high grade gliomas^42,45^. Apart from the differential H3K27 acetylation of the enhancers, we also uncovered that the poorly acetylated enhancers are characterized by high DNA methylation, suggesting that these two molecular mechanisms may both be involved in the regulation of the *EGFR* expression.

We also identified epigenetic regulation cues for a large group of *PCDHG* genes in gliomas. *PCDHGA* genes encode *PROTOCADHERINs* that are involved in cell–cell adhesion, boundary formation and maintenance, as well as morphogenesis in the developing brain^45^. Certain clusters of *PROTOCADHERINs*, alfa (*PCDHA*) and gamma (*PCDHG*) have been shown to be regulated in a stochastic manner in the developing human neurons^46^. The stochastic *PROTOCADHERIN* selection occurs at the level of neuronal progenitors and *PROTOCADHERIN* expression is reduced or silenced in adult brain neurons. Chromatin activation (observed as H3K4me3 mark depositions) of the *PROTOCADHERIN* promoters controls the stochastic regulation of their expression^46^. *PROTOCADHERIN* expression loss or reduction has been linked to poor prognosis in breast cancer patients^47^. DNA methylation at the *PROTOCADHERINs* promoters was observed to decrease their expression in many types of cancers^48^, including gliomas^6^. Here, we demonstrated the highest expression of the *PCDHG* genes in PA samples, moderate in DA and the lowest in GBM/pGBM. Those genes exhibited high correlation of transcriptional activity with H3K4me3 and H3K27ac deposition at their promoters, which is in concordance with previously reported results^46^. Moreover, we predicted contacts of those genes with enhancers located up to the 2 Mb away from their promoters, including a differentially acetylated (H3K27ac) one. H3K27ac deposition at the non-promoter regions is usually associated with active enhancers^18^. The highest H3K27 acetylation at enhancers contacting the *PCDHG* genes was found in PA and correlated well with their increased expression. As a result, we identified a new potential mechanism by which acetylation of the contacting enhancer regulates the expression of the *PCDHG* gene cluster. Long-range regulatory elements were earlier reported but only for *PCDHA* gene cluster^49^. To the best of our knowledge this is the first reported finding related to *PCDHG* cluster enhancer. In glioma DEGs, the *PCDHGA* gene cluster was also found within the most enriched TADs.

Next, we identified TFs negatively correlated with the *PCDHG* cluster genes expression, namely E2F1 and REST, known transcriptional repressors^50,51^. E2F1 is known to be upregulated in a variety of human cancers^52^ and we found that it was also upregulated in higher-grade gliomas. Furthermore, we identified a positive correlation between the expression of the *PCDHG* gene cluster and two other transcription factors, LMO2 and NR2F1, which can act as transcriptional activators or repressors depending on their interacting partners^53,54^. Intriguingly, the positive correlation was also found with KLF12 which was reported to act as a repressor^55^.

Overall, the results shown here demonstrate the existence of epigenetic differences associated with chromatin organization driving differential gene expression in gliomas of various malignancy. In low- and high-grade gliomas, we showed that combining the whole genome, high-throughput epigenetic data with Hi-C data and transcriptomic profiles can reveal new regulatory networks.

## Materials and methods

### Samples and wet lab experiments

Our analysis covered the following glioma samples: pilocytic astrocytomas (PA, WHO grade I, n = 11), diffuse astrocytomas (DA, WHO grade II or III, n = 7), glioblastomas (GBM, WHO grade IV, n = 14) and pediatric glioblastoma (pGBM, WHO grade IV, n = 1) with the following data: ATAC-seq, DNAse I-seq, H3K4me3, H3K27ac, H3K27me3 ChIP-seq, DNA methylation sequencing and RNA-seq (see Supplementary Information for details). The description of the laboratory procedures is available in the publication by Stępniak et al.^12^ and the processed data, ready for visualization in genome browsers, are available at http://regulomics.mimuw.edu.pl/GliomaAtlas/.

### Genes with differential expression / epigenetics marks deposition

Differentially expressed genes (DEGs) were determined using DESeq2^56^, with false discovery rate (FDR) correction for multiple testing and significance level threshold of FDR < 0.01. DEGs were estimated for three grade specific pairwise comparisons: PA vs DA, DA vs GBM/pGBM and PA vs GBM/pGBM. Next, because the DA samples consisted of IDH-mutant (n = 3) and IDH-wild type (n = 4) samples, we verified the influence of IDH status on identified DEGs. Hence, PA vs DA and DA vs GBM/pGBM comparisons were performed independently for DA IDH-mutants or DA IDH-wild type samples. The overlap of DEGs returned from two grade pairwise comparisons (PA vs DA, DA vs GBM/pGBM) with the entire set of DA samples and DA samples separated into two groups in respect to IDH gene status, was calculated. Moreover, because this study includes a limited number of samples, we also computed differentially expressed genes in gliomas of WHO grade II/III vs WHO grade IV in the TCGA dataset. The comparison was performed on DESeq2^56^ normalized fragment counts with Wilcoxon test followed by Bonferroni correction with corrected p value cut-off point equal to 0.01. The differential genes for GII/III and GIV obtained on both datasets were intersected to learn about the common part (see Supplementary Information for details).

Using DESeq2 with the same settings and significance level threshold of FDR < 0.01, there were identified genes with differential epigenetic marks (DEMs) deposited at their promoter regions defined as TSS ± 2 kb. As an input the NGS counts within promoters from the following assays were included: H3K4me3 and H3K27ac, ATAC-seq, DNA methylation-seq and DNAse I-seq. To identify genes with differential DNA methylation we used DESeq with the same procedure, but as an input we used the number of hypermethylated (beta value ≥ 0.8) cytosines in the CpG context. To obtain DNA methylation beta values from fastq data, the pipeline recommended by Roche^57^ was implemented into CytoMeth (https://github.com/mdraminski/CytoMeth) tool which was further extended. To determine methylation percentage, the CytoMeth tool calls the methratio.py script supplied by BSMAP^58^.

### Searching for correlation between H3K4me3 and H3K27ac marks and gene expression

Correlation of gene expression and epigenetic marks within the promoters of DEGs or genes with DEMs was calculated with Spearman correlation, across patients.

To compare correlation strength between gene expression and epigenetic marks among DEGs and randomly chosen active genes (≥ 10 reads on average in all samples), we selected sets of genes having on average the same expression levels as in the original set of DEGs and calculated correlations for these random genes. We used the canonical TSS for all of the genes in this study and promoters were defined as TSS ± 2 kB. To verify the significance of the overlap of DEGs and genes with DEMs, we calculated the probability of getting the original number of overlapping genes or greater with bootstrapping procedure (see Supplementary Information for details).

We compared Spearman correlations of DEGs with epigenetic marks (H3K27ac, H3K4me3) deposited within their promoters to correlations obtained for randomly paired non-DEGs with their matching epigenetic marks. The randomly selected non-DEGs were sampled from active genes under the condition that their mean expression level was at least the same as that of the DEGs (see Supplementary Information for details).

Then, for the DEGs having Spearman rho > 0.7 we estimated whether they are of prognostic importance for glioma patient survival, according to proteinatlas.org^59^ (Supplementary Dataset S1). The overlap between the highly correlating DEGs with protein atlas prognostic genes was divided by the total number of DEGs. Genes were estimated as having a prognostic value if they passed the log-rank test with p value below 0.001 using dataset (Supplementary Dataset S1) from proteinatlas.org^59^.

### Distribution of chromatin states across topologically associating domains

Genes genomic coordinates were taken from the genome-build-accession NCBI:GCA_000001405.20 (hg38) and gene promoters were defined as TSS ± 2kB. Next, deposition of H3K4me3, H3K27ac, ATAC-seq, DNAse I-seq and DNA methylation was assigned to such defined promoters and further to genomic coordinates of topologically associating domains (TADs). TADs segmentation (n = 3.165 TADs) and borders’ coordinates were derived from Hi-C data from a developing human cerebral cortex in midgestation^27^. To verify the co-regulation of gene expression and epigenetic marks in respect to TADs segmentation, we compared the log2 fold changes of counts from each of the high-throughput measurements obtained for each of the group pairwise comparisons (PA vs DA, DA vs GBM/pGBM, PA vs GBM/pGBM) with Kruskal–Wallis test. In the test, log2 fold changes were used as values and TAD borders as groupings. To verify its significance on real data, the permutation test was applied on the shuffled genes expressions/epigenetic marks among TADs, maintaining for each TAD its original number of genes (see Supplementary Information for details).

### Identification of TADs enriched for DEGs/DEMs

The TADs segmentation was acquired from the already published data where TADs borders’ coordinates have been computed based on Hi-C data^27^. Here, to the known TADs segments we assigned DEGs and genes with DEMs. Next, using a custom script, we verified which TADs were enriched for DEGs and DEMs. For each TAD we calculated the probability of obtaining the observed number of DEGs/DEMs or higher, using a pbinom function from R ‘stats’ package, with the following parameters: q = (number of DEG/DEMs − 1); size = number of genes in the TAD; prob = share of DEG/DEMs among all genes and lower.tail = F. TADs with fewer than three genes were excluded from the binomial distribution testing. The Benjamini–Hochberg procedure^60^ was used to correct the p-values. The entire procedure was repeated for all types of high-throughput measurements, and three grade-differential pairwise comparisons.

### Relationship between DEGs and epigenetic marks deposition in TADs

To find possible association between co-occurring changes in gene expression levels and H3K27ac/H3K4me3 depositions, we calculated for each TAD the mean gene expression fold changes of the genes present within a given TAD as well as the mean H3K27ac/H3K4me3 deposition fold changes. Finally, we correlated fold changes of gene expression levels within TADs across three grade-differential pairwise comparisons of grades with their matching epigenetic marks fold changes obtained for the same TADs. Enrichr^61^ was used to assign functions to genes found in the most enriched TADs.

### Copy number alterations analysis

In order to identify and visualize copy number variations (CNAs) in gliomas, we employed CaSpER^62^, a signal processing tool that uses RNA-seq information to detect focal and large-scale genetic variations. B-allele frequencies were generated from RNA-seq pileup data to decipher allelic imbalances in PA, DA and GBM samples. We also included one normal brain tumor sample as a control and computed large CNA events. Concurrently, NGS reads were summarized at the gene level using featureCounts, in both paired-end and reversely stranded modes^63^ and resulting raw data were normalized to TPMs. Following that, CNVs were identified using the CaSpER tool author's guidelines.

### Identification of TFs in genes regulatory regions

To identify TFs within regulatory regions we used DAVID tool with FDR < 0.01. Furthermore, prediction of TFs binding sites from DAVID tool was confirmed by an additional analysis of ATAC-seq data^12^ employing the BMO TF binding sites prediction tool—a method to predict TF binding sites without using footprints^28^ (see Supplementary Information for details). Next, the identified TFs were assigned to target gene/genes. We computed Spearman correlation between the expression of a gene coding a TF and its assigned target gene/genes. We also computed correlation of the same TFs with randomly selected active genes as target genes. Then, we considered only those TFs whose real target genes correlation was higher than the maximal correlation of TFs with randomly paired target genes (or lower than the minimal in the case of negative correlation).

### TADs with genes with bivalent chromatin

Due to sample material limitations, the identification of bivalent chromatin was performed only on GBM/pGBM samples. The only H3K27me3 ChIP-seq data of acceptable quality that we had at our disposal came from GBM/pGBM samples. To identify bivalent chromatin, we searched for DEGs from PA vs GBM/pGBM comparison with simultaneous presence of H3K4me3 and H3K27me3 marks at their promoters. We sorted those genes based on their H3K4me3 and H3K27me3 signals and chose 1000 with the highest signals in each group (resulting in > 27.000 counts per sample for H3K4me3 peaks; > 38.000 counts per sample for H3K27me3). Subsequently, DEGs from PA vs GBM/pGBM comparison with identified bivalent chromatin at their promoters were assigned to TADs. Then, we used the hypergeometric distribution test to calculate the likelihood of the obtained overlap among the 'glioma TADs' and TADs containing DEGs with bivalent chromatin.

### Long-range intra-chromosomal contacts of DEGs and enhancers

The genomic ranges of active enhancers were determined by the presence of H3K27ac peaks in non-promoter regions, (with promoters defined as TSS ± 2kB). To identify enhancers’ target genes we selected contacts between DEGs from each of the three grade-differential pairwise comparisons and enhancers within the 2 Mb range based on chromatin contact maps that have been generated by Won et al.^27^ from the Hi-C data from developing human brains.

### Enhancers with grade-specific activity

To detect grade specific enhancers the differentially acetylated enhancers and differentially methylated CpGs within enhancers were pairwise compared between grades and statistical significance was defined with Mann–Whitney-Wilcoxon test p value cutoff < 0.01. Gene set enrichment analysis (GSEA)^64^ was used to calculate enrichment scores for genes with multiple contacts with enhancers on the same chromosome and DEGs with at least one contact with differentially acetylated enhancers between PA and GBM/pGBM. Other GO terms enrichments were discovered using the DAVID tool^65^ (see Supplementary Information for details).

The Spearman correlation was computed between differentially acetylated enhancers and their target genes (n = 117). The permutation test was applied to test the significance of the correlation between DEGs expression and H3K27ac marks deposited at the contacting enhancers. For each grade separately we randomly paired DEGs expression levels and H3K27 acetylation at enhancers and calculated correlation. The procedure was repeated 100 times and means were computed each time. For each enhancer's correlation with DNA methylation, we used the average of CpG methylation levels within that enhancer.

### Patients’ survival analysis

Prognostic value of the *EGFR* gene expression for the patients’ survival was assessed using the Rembrandt repository—a large collection of genomic data from brain cancer samples^36^. Prognostic value of other genes contacting differentially acetylated and differentially DNA-methylated enhancers, for the patients’ survival was assessed based on the TCGA dataset (TCGA Research Network: https://www.cancer.gov/tcga). The analysis was done using the survival R package.

### Ethics approval

The tissue collection protocol was approved by the Committees of Bioethics of the neurosurgery clinics where tumour samples were obtained: The Children’s Memorial Health Institute, Public Central Clinical Hospital, Institute of Psychiatry and Neurology and Mazovian Brodno Hospital (protocol number #14/KBE/2012, #KBE/54/2016, #3/2016). Each patient gave informed consent to the use of tumour tissues, which were then anonymized. All experiments were performed in accordance with relevant guidelines and regulations.

## Supplementary Information

Supplementary Information 1.

Supplementary Information 2.

Supplementary Information 3.

Supplementary Information 4.

Supplementary Information 5.

Supplementary Information 6.

Supplementary Information 7.

Supplementary Information 8.

## Data Availability

Scripts written in R or python allowing to reproduce the results are deposited at https://github.com/ilona-grabowicz/epigenetics-in-glioma.
