# Author Correction: The role of epigenetic modifications, long-range contacts, enhancers and topologically associating domains in the regulation of glioma grade-specific genes

**DOI:** 10.1038/s41598-021-99319-4

**Published:** 2021-09-28

**Authors:** Ilona E. Grabowicz, Bartek Wilczyński, Bożena Kamińska, Adria‑Jaume Roura, Bartosz Wojtaś, Michał J. Dąbrowski

**Affiliations:** 1 Institute of Computer Science of the Polish Academy of Sciences, Warsaw, Poland; 2 Faculty of Mathematics, Informatics and Mechanics, University of Warsaw, Warsaw, Poland; 3 Nencki Institute of Experimental Biology of the Polish Academy of Sciences, Warsaw, Poland

Correction to: *Scientific Reports* 10.1038/s41598-021-95009-3, published online 02 August 2021

The original version of this Article contained an error in Figures 1 and 2 where the colour key was missing in panels 1C, 1D, and 2E. The original Figures 1 and 2 and accompanying legends appear below.

**Figure 1:**
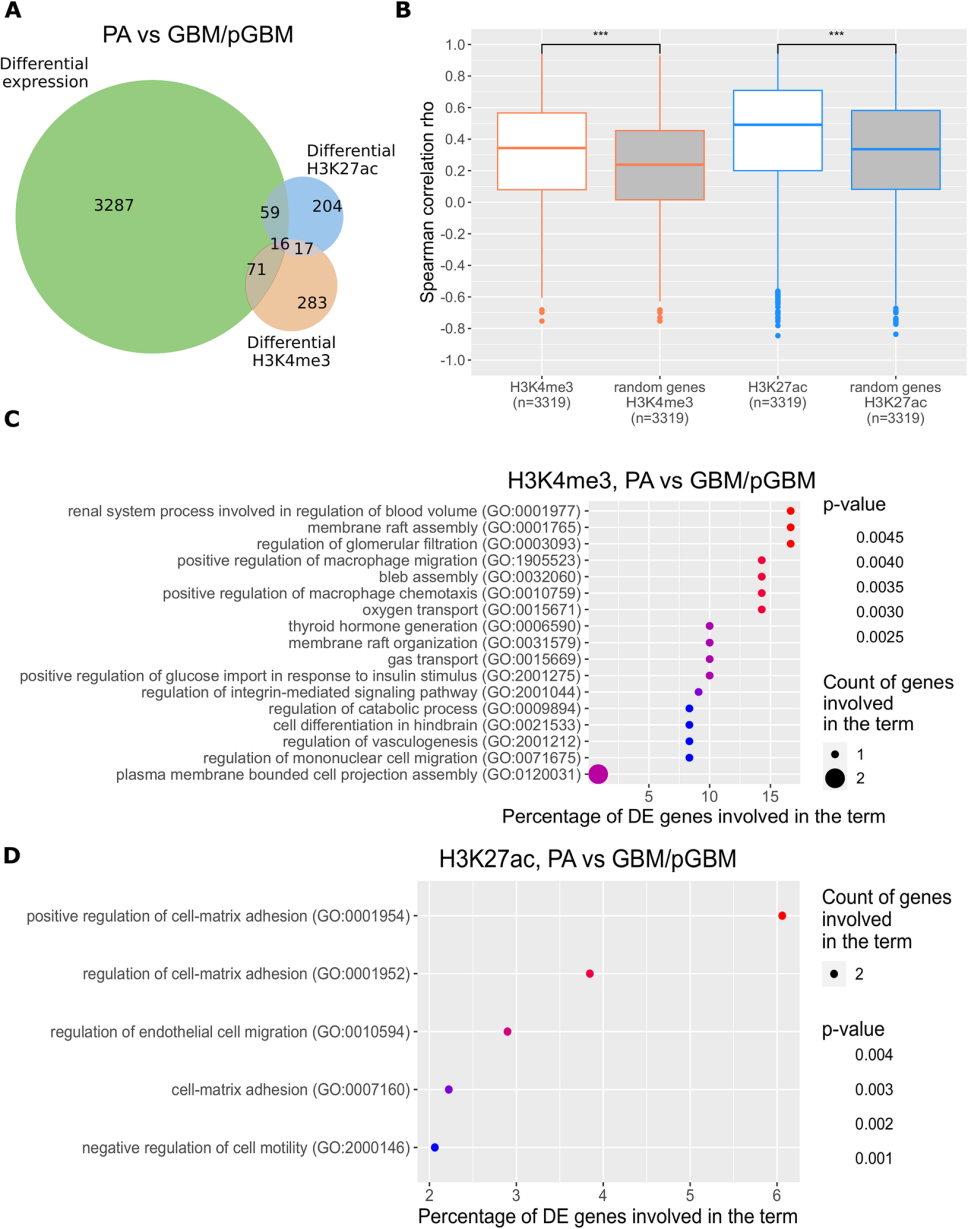
Identification of genes differentially expressed in benign and malignant gliomas (PA vs GBM/pGBM). (**A**) Intersection of differentially expressed genes (DEGs) with genes carrying differential epigenetic modifications (DEMs) for the PA vs GBM/pGBM comparison. (**B**) Correlation of H3K4me3 (orange boxes) and H3K27ac (blue boxes) coverages at the promoters of DEGs (PA vs GBM/pGBM) with their expression. Boxes filled with white show values for DEGs, while in grey for the randomly chosen active genes. (**C**) Enrichment of Biological Process GO terms for DEGs in PA vs GBM/pGBM comparison having high correlation of expression levels with H3K4me3 (Spearman rho > 0.7), and being prognostic for glioma patients’ survival (log-rank test p < 0.001). (**D**) As in (**C**) for H3K27ac.

**Figure 2:**
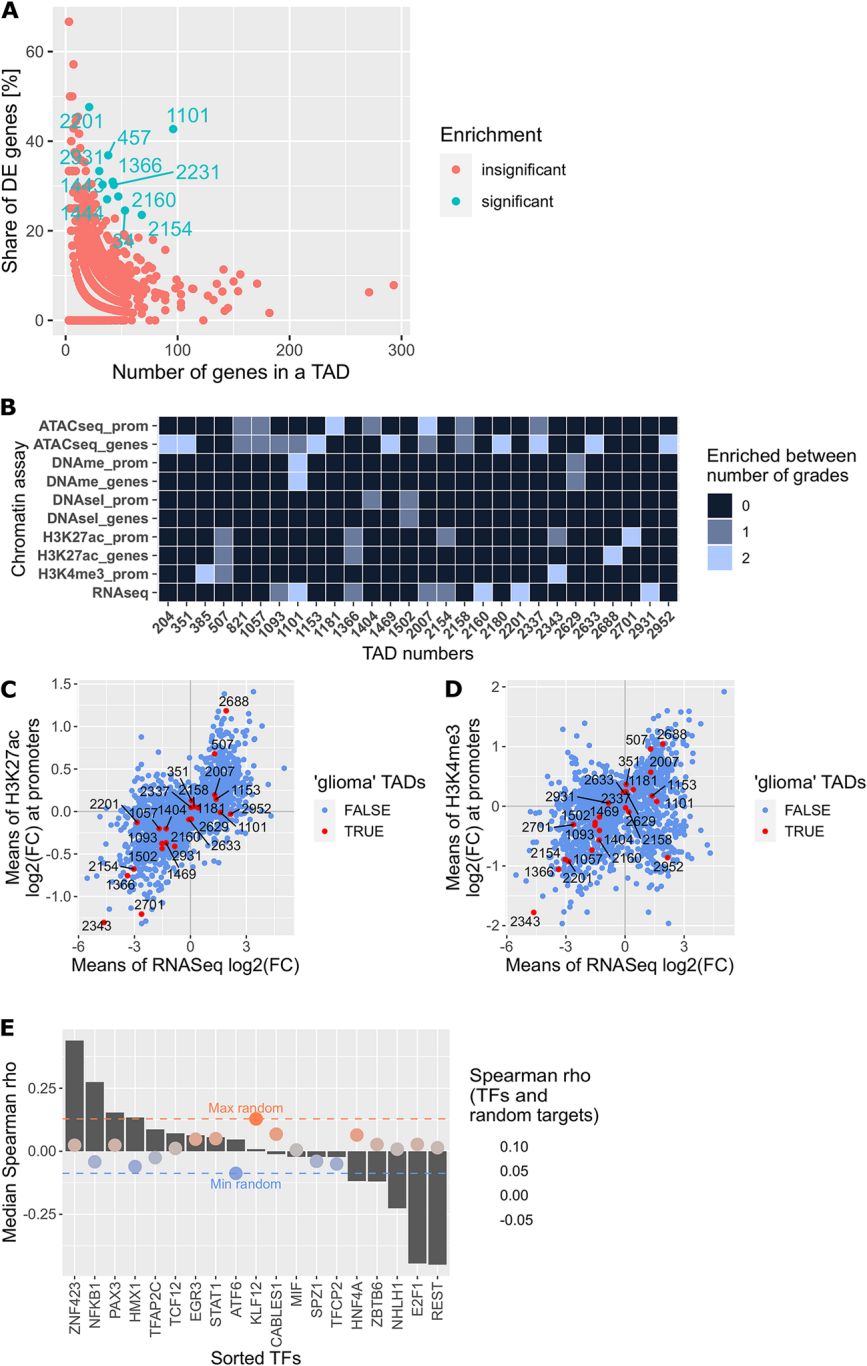
Discovering TADs enriched in genes differentially expressed and epigenetically modified in gliomas of different malignancies. (**A**) TADs enriched for DEGs or genes with DEMs deposited at their promoters, in the PA vs GBM/pGBM comparison. (**B**) TADs with exceptionally high proportion of DEGs and genes carrying DEMs in all grades comparisons. Colour scale depicts in how many of the three grade-comparisons a particular TAD was found to be enriched. (**C**) Dotplot showing means of DEGs expression and H3K27ac peak signals fold changes between PA and GBM/pGBM samples. Each dot represents a mean for each TAD. Dots in red mark the most enriched TADs (‘glioma TADs’, binomial test Benjamini–Hochberg corrected p < 0.05). (**D**) As in Fig. 2C for H3K4me3. (**E**) Spearman correlation between gene expression levels of enriched TFs and their target genes within TAD 2337. Grey bars depict median Spearman correlation between expression levels of genes encoding TFs and their target genes. Red and blue lines demarcate maximal and minimal correlation between expression levels of TF-coding genes and randomly selected, active genes. Colours of dots show median level of Spearman correlation between TFs and random target genes (red—positive correlation, blue—negative correlation).

The original Article has been corrected.

