# Supplementary material for: The role of epigenetic modifications, long-range contacts, enhancers and topologically associating domains in the regulation of glioma grade-specific genes
Source: Sci Rep. 2021 Aug 2;11:15668. doi: 10.1038/s41598-021-95009-3 (PMC8329071; doi:10.1038/s41598-021-95009-3)
Supplement: Supplementary file 8 — Supplementary Information 8. [file 41598_2021_95009_MOESM8_ESM.pdf]

The role of epigenetic modifications, long-range contacts, enhancers and topologically associating domains in the regulation of glioma grade-specific genes

Ilona E. Grabowicz[1*], Bartek Wilczyński[2], Bożena Kamińska[3], Adria-Jaume Roura[3], Bartosz Wojtaś[3] and Michał J. Dąbrowski[1*]

[1] Institute of Computer Science of the Polish Academy of Sciences, Warsaw, Poland

[2] Faculty of Mathematics, Informatics and Mechanics, University of Warsaw, Warsaw, Poland

[3] Nencki Institute of Experimental Biology of the Polish Academy of Sciences, Warsaw, Poland

*Corresponding authors

E-mail: ilona.grabowicz@gmail.com (IEG), m.dabrowski@ipipan.waw.pl (MJD)

Supplementary Information

Results

Correlation of fold changes levels of ChiP-seq marks with DEGs expression at the TADs level

For TADs containing DEGs between PA vs GBM/pGBM, Spearman rho correlation coefficient for H3K27ac was 0.66 (p = 8.7 e-203). For the selected ‘glioma TADs’ the correlation was even stronger: Spearman rho = 0.86, (p = 2e-6). Similarly, rho = 0.31 for H3K4me3 and all TADs (p = 6.8e-37), but rho = 0.75 for ‘glioma TADs' (p = 4e-5).

Methods

Samples and wet lab experiments

In our study, we analysed data from Stępniak et al.[1] on glioma samples which included gliomas assigned to different malignancy groups: pilocytic astrocytomas (PA, WHO grade I, n = 11), diffuse astrocytomas (DA, WHO grade II or III, n = 7), glioblastomas (GBM, WHO grade IV, n = 14) and pediatric glioblastoma (pGBM, WHO grade IV, n = 1). PA and pGBM samples were derived from pediatric patients. We studied the chromatin openness state (using ATAC-seq, DNAse I-seq), deposition of epigenetic marks characteristic for open (H3K4me3, H3K27ac) or closed chromatin (H3K27me3) using ChIP-seq assays and DNA methylation (whole genome bisulfite sequencing) in the glioma samples as well as gene expression (RNA-sequencing) in freshly isolated gliomas of various grades.

Genes with differential expression / epigenetics marks deposition

Differentially expressed genes (DEGs) were determined using DESeq2[2], with false discovery rate (FDR) correction for multiple testing and significance level threshold FDR<0.01. Differential expression analysis with DESeq2 was performed for three pairwise comparisons: i) WHO GI pilocytic astrocytomas (PA) vs WHO GII/GIII diffuse astrocytomas (DA) (PA vs DA), ii) PA vs WHO GIV glioblastomas (GBM) and pediatric glioblastoma (pGBM) (PA vs GBM/pGBM) and iii) DA vs GBM/pGBM. Genes with differential epigenetic marks (DEMs) were identified using DESeq2 and FDR<0.01 in the same way as DEGs. Using various assays, we identified genes having grade-differential signals at their promoters or gene bodies. For promoters, which were defined as TSS ± 2kB, we used data from H3K4me3 and H3K27ac, ATAC-seq, DNA methylation and DNAse I-seq assays. For gene bodies, we used DNA methylation assays. DEGs and genes with assigned DEMs were chosen for further steps in our analytical pipeline.

Testing the significance of the overlap between genes with differential expression and differential deposition of the epigenetic marks

To verify the significance of the overlap of DEGs and genes with DEMs, we performed bootstrapping. From a pool of active genes (defined as those with a mean raw count across samples > 10), genes matching the number of: (1) obtained DEGs and (2) obtained genes with DEMs we randomly selected, and the overlap between them was calculated. Then, a random selection out of all, active genes of two sets of genes matching numbers of DEGs and genes with DEMs was repeated 100 times. In the next step, we calculated the number of times the overlap of the randomly selected DEGs and genes with DEMs was equal to or greater than the number of the originally overlapping genes.

Searching for correlation between H3K4me3 and H3K27ac marks and gene expression

In the case of DEGs, the Spearman correlation between expression levels and H3K4me3 and H3K27ac levels was calculated across samples. We compared the obtained correlation for DEGs with their epigenetic marks to randomly selected non-DEGs with their matching epigenetic marks. The randomly selected non-DEGs were sampled from active genes (average expression levels $\geq$ 10 raw counts across samples) under the condition that their mean expression level was at least the same as that of the DEGs, to avoid possible bias resulting in stronger correlation due to stronger expression. Correlation was calculated across all samples of our dataset, separately for each of the pairwise comparisons: PA vs DA, PA vs GBM/pGBM, DA vs GBM/pGBM. Random selection of active genes matching numbers of each of the two groups was repeated 10 times for each epigenetic mark and grade pairwise comparisons.

Distribution of chromatin states across topologically associating domains

All genes' genomic coordinates (defined as the region from gene start to gene end) were obtained from the human reference genome genome-build-accession NCBI:GCA_000001405.20 (hg38). Promoter regions were defined as ± 2kB from TSS. For so defined promoters, data on deposition of H3K4me3, H3K27ac, open chromatin (ATAC-seq and DNAse I-seq) and DNA methylation were assigned. Genes and their epigenetic marks were assigned into TADs genomic coordinates using Hi-C data[3]. To test co-regulation of different epigenetic marks and gene expression within TADs, we used the Kruskal-Wallis test. As the input for Kruskal-Wallis test we used the log2 fold changes of gene expressions or fold changes of the epigenetic marks depositions. Those fold changes were calculated using DESeq2. PA and DA, PA and GBM/pGBM, and DA and GBM/pGBM were all compared using DESeq2. In the Kruskal-Wallis test, calculated log2 fold changes were used as values and TAD borders

as groupings. We used the fold changes of counts in promoter regions (TSS ± 2kB) in case of H3K4me3 and H3K27ac, DNA methylation, ATAC-seq and DNAse I-seq, as well as counts from the gene bodies for DNA methylation, ATAC-seq and DNAse I-seq. To verify the significance of the Kruskal-Wallis test performed on real data, we applied permutation tests. Data from each of the high-throughput measurements and within each of the group pairwise comparisons were subjected to a permutation test. More specifically, we shuffled the genes expression values / epigenetic marks values among the TADs, maintaining the original numbers of genes in each TAD. We performed the permutations 10 times for each type of data (e.g., ATAC-seq-gene bodies) and grade comparison. The Kruskal-Wallis H statistics and p values were calculated for all of the tests. The original H statistics and p values were always at the top (or bottom, respectively) of the ranked, permuted values.

Identification of TFs potentially regulating DEGs present within the 'glioma TADs' and enhancers

Additionally, prediction of TFs binding sites from DAVID tool was confirmed by an additional analysis of ATAC-seq data[4] employing the BMO tool - a method to predict TF binding sites without using footprints. First, low-quality reads and PCR duplicates were removed and only properly paired and uniquely mapped reads were retained for downstream analysis. Next, ATAC-seq peaks were called using MACS2[5] using "broad" and "nomodel --shift -100 --extsize 200" flags. Resulting peaks were then intersected with human ENCODE blacklist regions (hg38) to discard the peaks within artifact regions. Concurrently, FIMO tool[6] was used to predict occurrence of known motifs across the human genome (fasta file). Altogether, BMO pipeline was run and only the significant binding motifs instances, based on adjusted Benjamini-Yekutieli test (-log10 adjusted p-value < 0.05), were selected.

For the 315 genes encompassed within 'glioma TADs' (Supplementary Table S2) we specified which TFBS are present within their promoters using the BMO tool. Gene promoters were defined as a sequence of 2 kB ± from TSS. Detection of TFBS with BMO was performed using ATAC-seq from eight tumour samples (PA n=4, DA n=2, GBM n=2). TFBS obtained with BMO allowed us to select TFs which could potentially regulate the expression of the genes of interest. Finally, using BMO, TFBS were also detected in the enhancer region (chr5:141528260-141529747) associated with *PCDHGA* genes cluster.

Long-range intra-chromosomal contacts of DEGs and enhancers

Following Johnston et al.[7] discovery that genes active in glioma stem cells with multiple loops are usually expressed at higher levels, we tested whether a similar pattern could be found in bulk tumour samples. Identification of enhancers, long-range contacts between genes and enhancers, as well as DNA methylation levels have all been previously described[4]. In brief, active enhancers were determined by the presence of H3K27ac peaks in non-promoter regions, (with promoters defined as TSS ± 2kB). We selected contacts between DEGs and enhancers within the 2 Mb range based on chromatin contact maps generated from the Hi-C data from developing human brains[3]. The differentially acetylated enhancers and differentially methylated CpGs within enhancers were defined by Mann-Whitney-Wilcoxon test p-value cut-off < 0.01. Enrichment scores for genes having multiple contacts with enhancers on the same chromosomes and for DEGs having at least 1 contact with differentially acetylated enhancers between PA and GBM/pGBM were calculated using gene set enrichment analysis (GSEA)[8]. Other enriched GO terms were identified using the DAVID tool[9]. GO terms enrichment threshold was p<0.005 with GO_Biological_Process_2018 and Enrichr algorithm enrichment. Results were visualized with the enrichR R package[10]. Calculations of correlation, statistical tests and permutation testing were performed using R (version 3.4). Genes for GSEA were ranked according to their number of loops.

Supplementary Figures

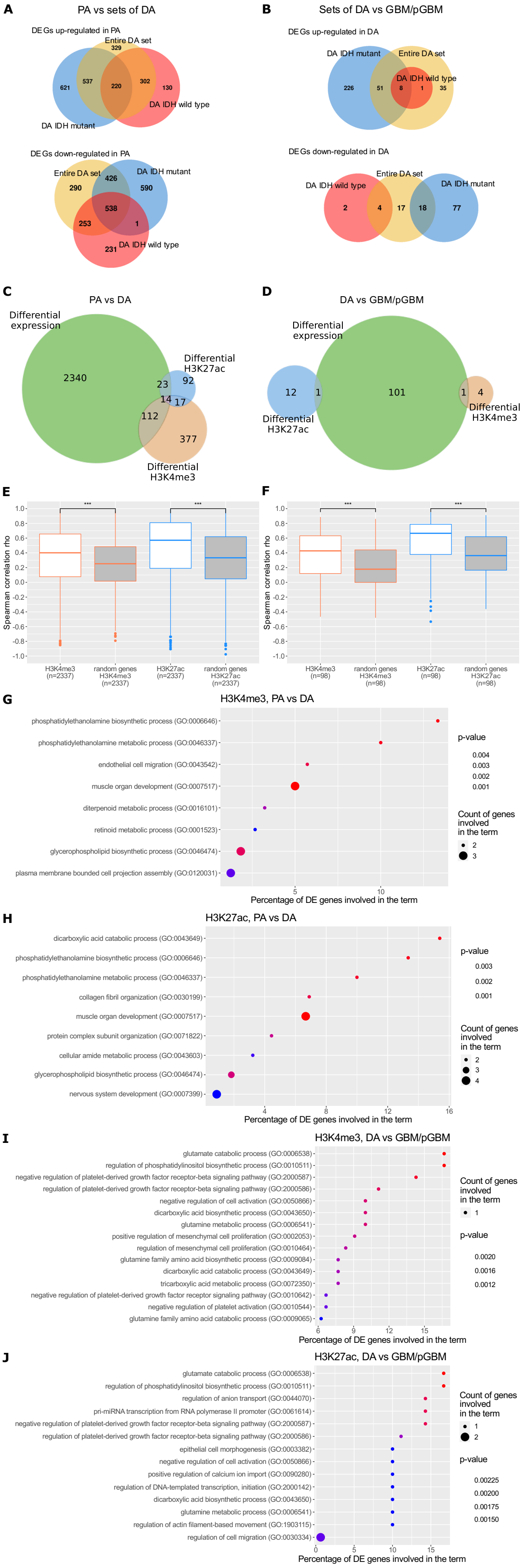

Supplementary Figure S1. Identification of differentially expressed genes (DEGs) in gliomas of different malignancy grades - comparison of PA vs DA and DA vs GBM/pGBM. **(A, B)** Overlap of down- and up-regulated DEGs identified in grade pairwise comparisons with entire set of DA samples and DA samples separated into IDH-status dependent groups. **(A)** Overlap of DEGs from DESeq2 analysis of PA vs DA, PA vs DA IDH-mutant and PA vs DA IDH-wild type samples. **(B)** Overlap of DEGs from DESeq2 analysis of DA vs GBM/pGBM, DA IDH-mutant vs GBM/pGBM and DA IDH-wild type vs GBM/pGBM samples. **(C)** Intersection of differentially expressed genes (DEGs) with genes carrying differential epigenetic modifications (DEMs) for the PA vs DA comparison. **(D)** As in Figure S1C, for DA vs GBM/pGBM comparison. **(E)** Correlation of H3K4me3 (orange boxes) and H3K27ac (blue boxes) coverages at the promoters of DEGs (PA vs DA) with their expression. Boxes filled with white show values for DEGs, while in grey for the randomly chosen active genes. **(F)** As in Figure S1E, for DA vs GBM/pGBM comparison. **(G)** Biological Process (GO) enrichment terms for DEGs in PA vs DA comparison with high correlation of expression levels with H3K4me3 (Spearman rho > 0.7), and being prognostic for glioma patients' survival (log-rank test < 0.001). **(H)** As in Figure S1G, for H3K27ac. **(I)** Enrichments for Biological Process GO terms for DEGs in DA vs GBM/pGBM comparison having high correlation of expression levels with H3K4me3 (Spearman rho > 0.7), and being prognostic for glioma patients' survival (log-rank test < 0.001). **(J)** As in Figure S1I, for H3K27ac.

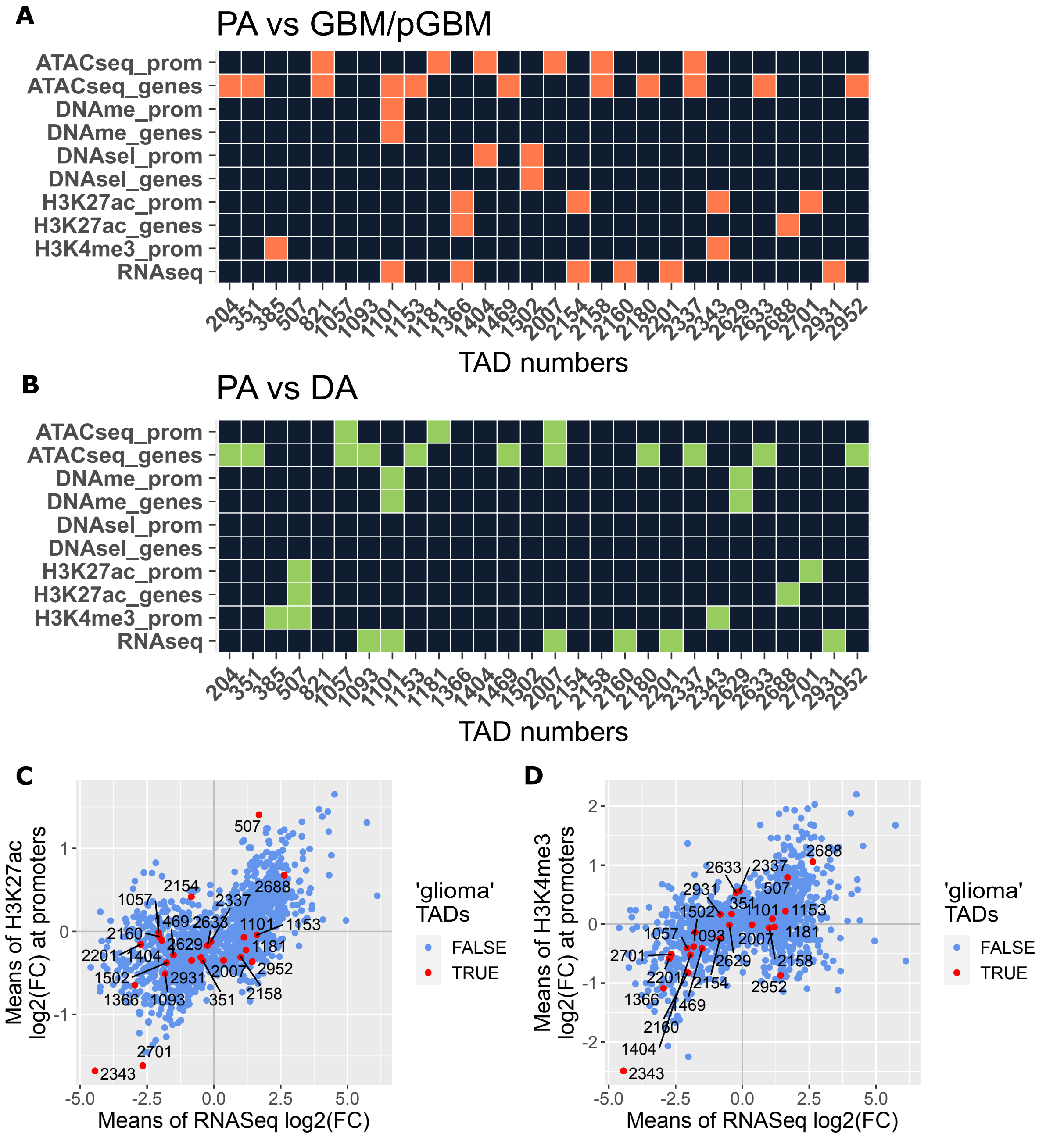

Supplementary Figure S2. **(A)** TADs with exceptionally high proportion of DEGs and genes carrying DEMs in the PA vs GBM/pGBM comparison. Color scale depicts in how many of the three grade-comparisons a particular TAD was found to be enriched. **(B)** As in Figure S2A, for PA vs DA comparison. **(C)** Dotplot showing means of DEGs expression and H3K27ac peak signals fold changes between PA and DA samples. Each dot represents a mean for each TAD. Dots in red mark the most enriched TADs ('glioma TADs', binomial test Benjamini-Hochberg corrected $p < 0.05$). **(D)** As in Figure S2C, for H3K4me3.

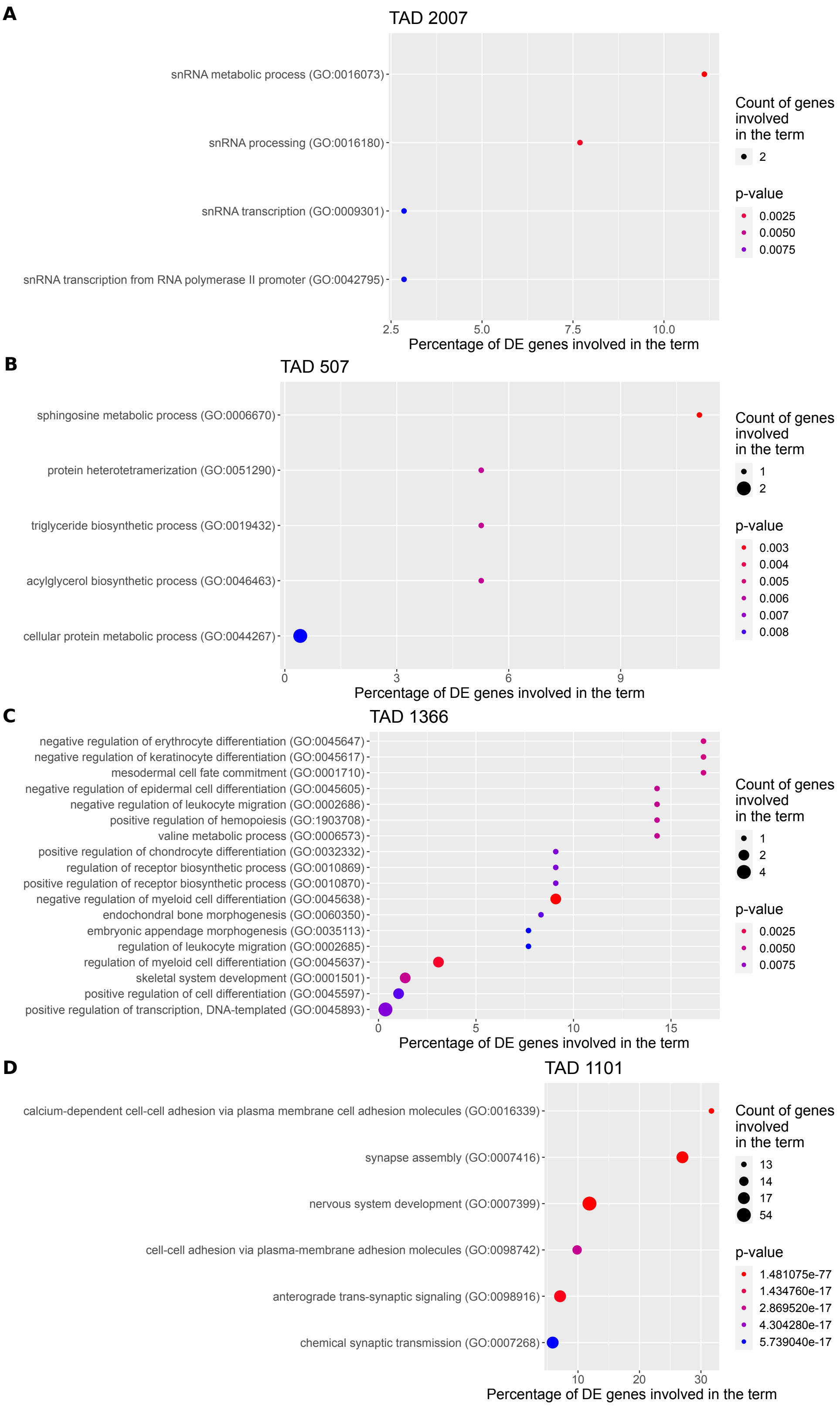

Supplementary Figure S3. Gene Ontology analysis of TADs with the high proportion of genes differentially active between different grades of gliomas. Enriched GO terms Biological Process categories for genes present within following TADs: (**A**) TAD number 2007. **(B)** TAD number 507. **(C)** TAD number 1366. **(D)** TAD number 1101.

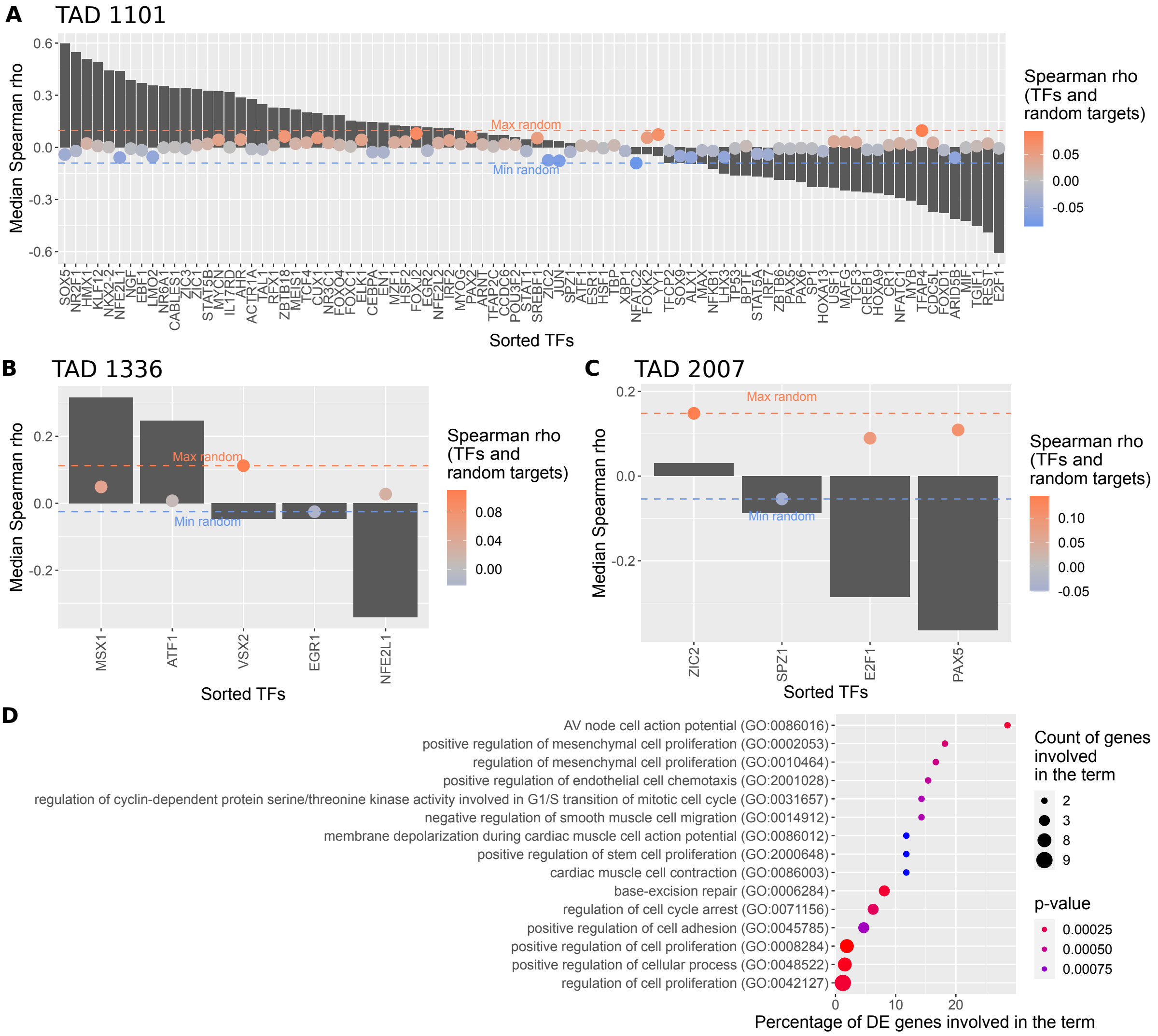

Supplementary Figure S4. Characterization of 'glioma TADs' transcriptional regulation by transcription factors and functional annotation of bivalent chromatin genes. Spearman correlation between gene expression levels of enriched TFs and their target genes within the following TADs: **(A)** TAD number 1101. **(B)** TAD number 1336. **(C)** TAD number 2007. Grey bars depict median Spearman correlation between expression levels of genes encoding TFs and their target genes. Red and blue lines demarcate maximal and minimal correlation between expression levels of TF-coding genes and randomly selected, active genes. Colours of dots show median level of Spearman correlation between TFs and random target genes (red - positive correlation, blue - negative correlation). **(D)** Enriched GO terms Biological Process categories for genes having bivalent chromatin.

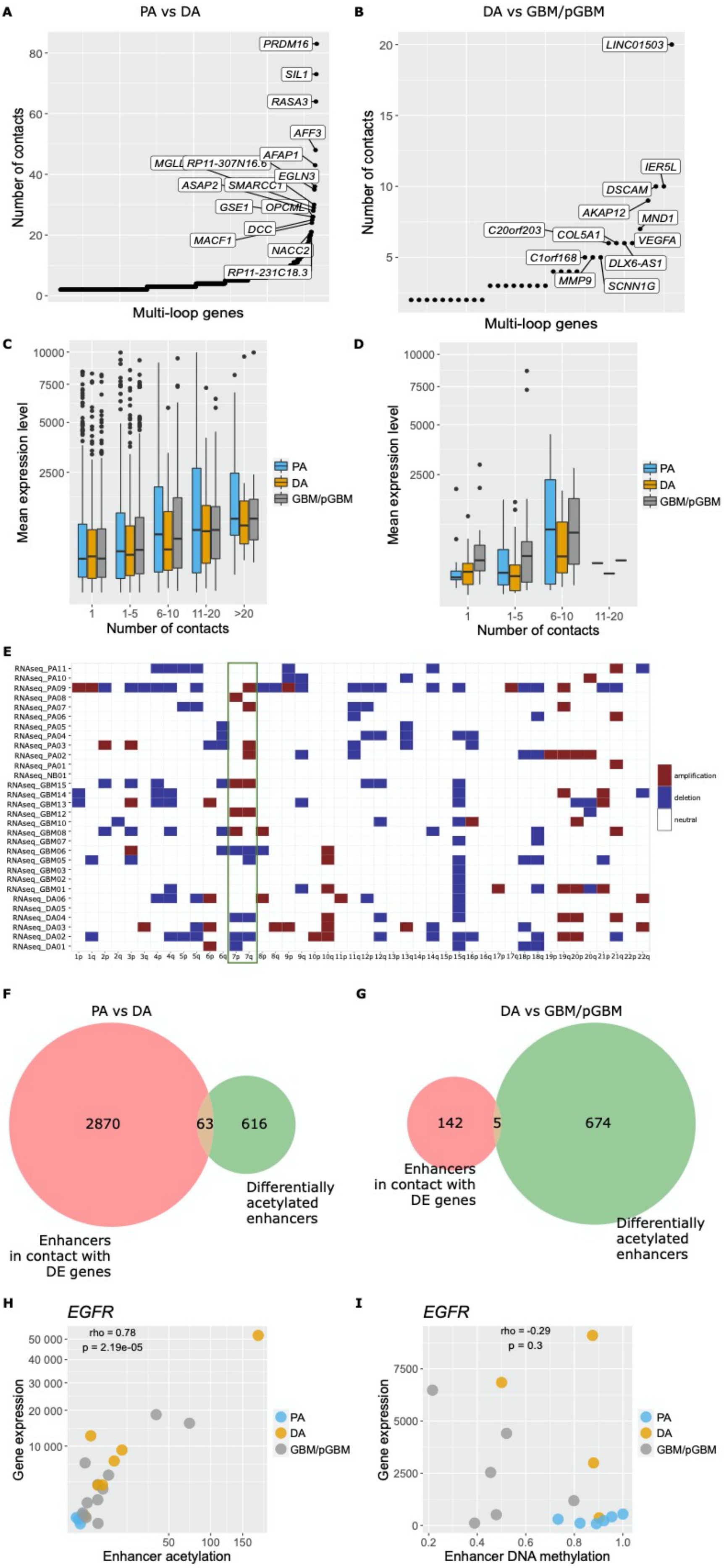

A
PA vs DA
Number of contacts
Multi-loop genes
PRDM16
SIL1
RASA3
AFF3
AFAP1
MGLL
RP11-307N16.6
EGLN3
ASAP2
SMARCC1
GSE1
OPCML
DCC
MACF1
NACC2
RP11-231C18.3
B
DA vs GBM/pGBM
Number of contacts
Multi-loop genes
LINC01503
IER5L
DSCAM
AKAP12
MND1
C20orf203
COL5A1
VEGFA
C1orf168
DLX6-AS1
MMP9
SCNN1G
C
Mean expression level
Number of contacts
1
1-5
6-10
11-20
>20
PA
DA
GBM/pGBM
D
Mean expression level
Number of contacts
1
1-5
6-10
11-20
PA
DA
GBM/pGBM
E
amplification
deletion
neutral
F
PA vs DA
2870
63
616
Enhancers in contact with DE genes
Differentially acetylated enhancers
G
DA vs GBM/pGBM
142
5
674
Enhancers in contact with DE genes
Differentially acetylated enhancers
H
EGFR
rho = 0.78
p = 2.19e-05
Gene expression
Enhancer acetylation
PA
DA
GBM/pGBM
I
EGFR
rho = -0.29
p = 0.3
Gene expression
Enhancer DNA methylation
PA
DA
GBM/pGBM

Supplementary Figure S5. Genes with multiple long-range contacts and enhancers targeting multiple genes - with the *PROTOCADHERINs* example and verification of CNA events. **(A)** DEGs from PA vs DA comparison with predicted multiple contacts with enhancers. **(B)** DEGs from DA vs GBM/pGBM comparison with predicted multiple contacts with enhancers. **(C)** Higher numbers of contacts with enhancers co-exist with higher expression of DEGs (PA vs DA). **(D)** As in Figure S5C, for DEGs of DA vs GBM/pGBM. **(E)** Visualization of the large-scale CNA events among all the samples. The chromosome 7p and 7q arms are highlighted, and amplifications, deletions, and neutral calls are color-coded. **(F)** Enhancers contacting DEGs (PA vs DA) intersect with the differentially acetylated (H3K27ac) (PA vs DA). **(G)** As in Figure S5F, for DA vs GBM/pGBM comparison. **(H)** Dotplot illustrating EGFR expressions in patients of all glioma grades vs contacting enhancer H3K27 acetylation. (**I**) Dotplot illustrating EGFR expressions in patients of all glioma grades vs contacting enhancer DNA methylation.

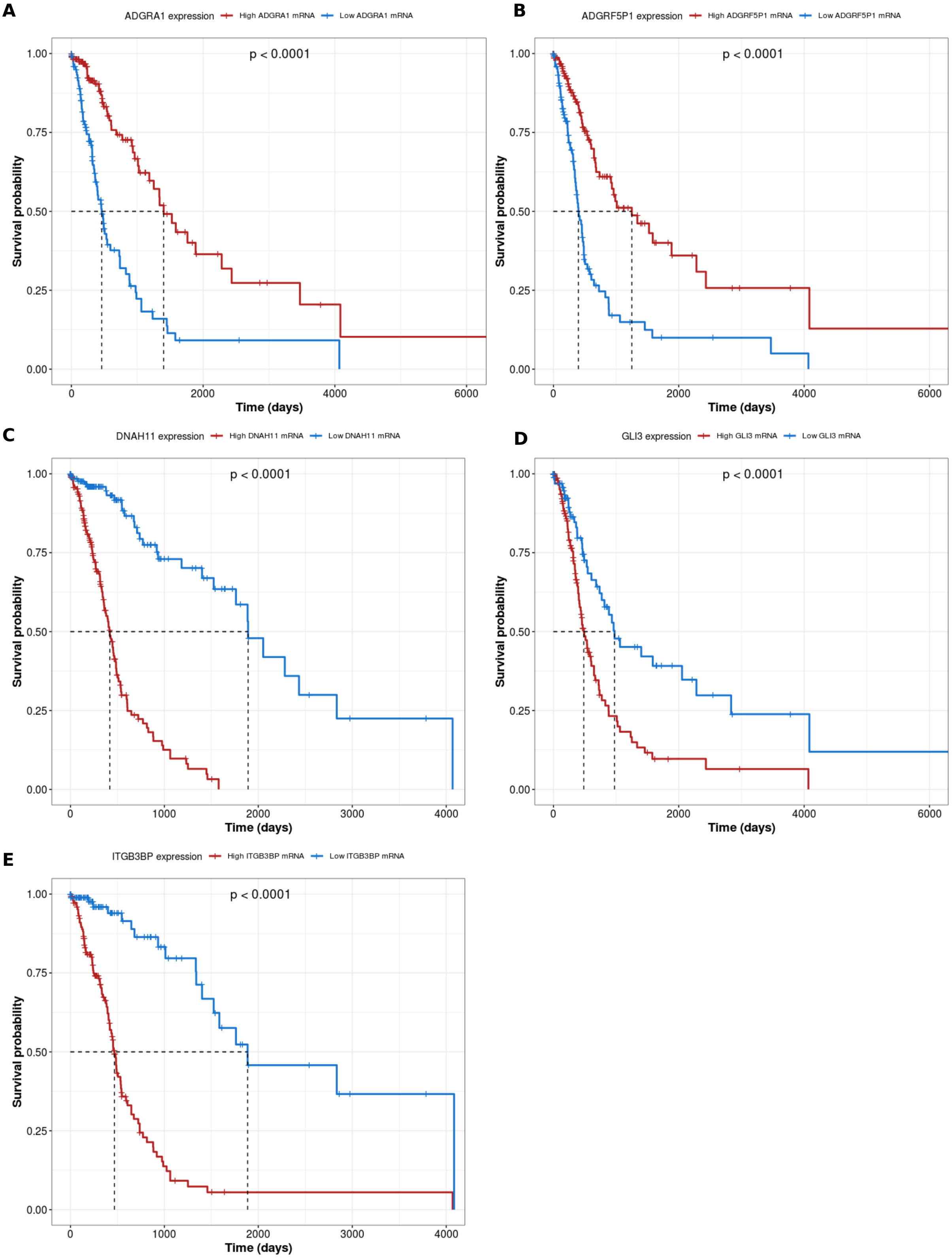

Supplementary Figure S6. Patients survival analysis for genes having differential enhancer H3K27 acetylation and DNA methylation between different glioma grades. Kaplan–Meier survival curves based on WHO GIII and GIV patients, data from TCGA. High gene expression is indicated in red while low gene expression is indicated in blue for the following genes: **(A)** *ADGRA1* **(B)** *ADGRF5P1* **(C)** *DNAH11* **(D)** *GLI3* **(E)** *ITGB3BP*.

Supplementary Tables

Supplementary Table S1. DEGs with expression levels correlating with H3K4me3 or H3K27ac at the level of >0.7 Spearman rho and being highly prognostic for glioma patients' survival (p<0.001 log-rank test, S1 Dataset).

Assay, comparison	DEGs, correlation>0.7, prognostic for patients' survival
H3K4me3, PA vs DA	*SNX10, PHGDH, QPRT, CPQ, AEBP1, ADAMTSL4, UBXN10, EN2, CARD19, HTRA1, ISG20, GPC5, MKRN3, ACHE, MXRA5, KCNN4, RARRES2, LOXL2, MSTN, ETNK2, RNF175, FBXO27, CYGB, RPL39L, ETNPPL, FOSL1, HIST3H2A, CASC10, EMP2, FBXO17*
H3K4me3, PA vs GBM/pGBM	*CPQ, UBXN10, MXRA5, RARRES2, SYT5, CYGB, CEND1, EMP2*
H3K4me3, DA vs GBM/pGBM	*ISG20, GLUD1, PDGFA*
H3K27ac, PA vs DA	*MTHFD2, SNX10, PHGDH, QPRT, CPQ, AEBP1, LOXL1, DNAJA4, CTF1, CRELD1, EN2, CARD19, SMPD1, ISG20, MKRN3, ACHE, RARRES2, LOXL4, MSTN, ETNK2, RNF175, GLUD1, FBXO27, CYGB, ETNPPL, C11orf24, GRIK1, HIST3H2A, RP11-195F19.5, CASC10, FBXO17*
H3K27ac, PA vs GBM/pGBM	*RWDD2A, SPAG4, QPRT, PDLIM2, HS3ST2, REEP2, ITPKA, RABGEF1, UBXN10, HTRA1, ACHE, IMPDH1, RARRES2, STAC2, THY1, STC1, PRSS12, C11orf24, CEND1, EMP2*
H3K27ac, DA vs GBM/pGBM	*ISG20, GLUD1, STC1, FOSL1, PDGFA*

Supplementary Table S2. Kruskal-Wallis test results for the original and permuted values of log2 fold changes between PA and GBM/pGBM samples grouped into TADs, for different datasets.

type of data	Kruskal-Wallis test H-statistic	p value	permuted 10x H-statistic (average)	permuted 10x p value (average)
H3K27ac (promoters)	6138	$5.0\ e^{-249}$	2813	0.46
ATAC-seq (promoters)	5304	$8.0\ e^{-157}$	2780	0.62
DNA-me gene body	5033	$5.5\ e^{-133}$	2807	0.42
H3K4me3 (promoters)	4864	$5.6\ e^{-114}$	2776	0.64
RNA-seq	4795	$8.2\ e^{-110}$	2783	0.56
DNAse I-seq (promoters)	4433	$6.7\ e^{-77}$	2784	0.61
DNA-me promoters	4193	$6.1\ e^{-70}$	2711	0.54

Supplementary Table S3. Kruskal-Wallis test results for the PA and DA samples. Statistical test results for the original and permuted values of log2 fold changes between PA (WHO GI) and DA (WHO GII/III) samples, grouped into TADs, for various high-throughput measurements.

Type of data	Kruskal-Wallis test H-statistic	p value	average H-statistic from 10 permutations	average p value from 10 permutations
H3K27ac (promoters)	5893	5.10 e^{-208}	2591	0.50
ATAC-seq (promoters)	5569	4.6 e^{-173}	2569	0.58
DNA-me gene body	4662	5.4 e^{-89}	2562	0.52
H3K4me3 (promoters)	5024	3.1 e^{-119}	2601	0.46
RNA-seq	4633	1.8 e^{-88}	2503	0.57
DNAse I-seq (promoters)	4022	7.1 e^{-41}	2600	0.46
DNA-me promoters	4701	2.1 e^{-98}	2506	0.37

Supplementary Table S4. Kruskal-Wallis test results for the DA and GBM/pGBM samples. Statistical test results for the original and permuted values of log2 fold changes between DA (WHO GII/III) and GBM/pGBM (WHO GIV) samples, grouped into TADs, for various high-throughput measurements.

Type of data	Kruskal-Wallis test H-statistic	p value	average H-statistic from 10 permutations	average p value from 10 permutations
H3K27ac (promoters)	6518	5.0 e^{-280}	2604	0.43
ATAC-seq (promoters)	6614	3.0 e^{-292}	2589	0.48
DNA-me gene body	4015	3.1 e^{-41}	2543	0.65
H3K4me3 (promoters)	5656	3.3 e^{-182}	2567	0.61
RNA-seq	4617	4.5 e^{-85}	2559	0.4
DNAse I-seq (promoters)	6246	3.1 e^{-248}	2563	0.62
DNA-me promoters	3683	8.1 e^{-26}	2512	0.50

Supplementary Table S5. Characterization of 'glioma TADs'. TADs enriched in at least 3 experiments for grade-differential comparisons (Fig. 2B). Functional annotation of genes identified in the 'glioma TADs' as well as correlation between TFs found within the promoters and their target DEG/DEGs.

TAD number	Biological Process GO terms	Enriched TFs common for the glioma TADs, (sign of correlation with target DEGs mRNA)
507<br>chr2:<br>221935281 -<br>222855282	sphingosine metabolic process	
1101<br>chr5: 140660415 -<br>141580433	synapse assembly, cell-cell adhesion<br>(*PcdhgA* genes)	*E2F1* (negative), *REST* (negative), *PAX5* (negative), *NFE2L1 (positive), NFKB1* (negative), *HMX1* (positive), ZBTB6 (negative)
1366<br>chr7: 27120381 -<br>28080381	cell differentiation<br>*(HoxA* genes)	*NFE2L1* (negative)
2007<br>chr11: 62312528 -<br>62872528	snRNA processing	*E2F1* (negative), *PAX5* (negative)
2337<br>chr14: 23170791 -<br>24410794	(cardiac) tissue morphogenesis, G-protein coupled receptor signaling	*E2F1* (negative), *REST* (negative), *NFKB1* (positive), *HMX1* (positive), *ZBTB6* (negative)
2343<br>chr14: 28650794 -<br>29490794	-	

Supplementary Table S6. Characterization of individual TADs. TAD number, range (chromosome, start, end), means and medians of gene expressions and ENSEMBL gene IDs of all genes assigned to the specific TAD are all provided for each TAD (.xlsx file).

Supplementary Table S7. TADs coordinates intersected with segment-level CNA calls. The width column indicates the length of a TAD. Only consistent CNA events (counts > 6) are provided, as suggested by the CaSpER methods. The letters p (short arm) and q (long arm) indicate the location of segment-based CNA (.xlsx file).

Supplementary Table S8. Association of TFs with genes assigned to the top six 'glioma TADs'. For differentially expressed genes within each of 'glioma TADs', the set of enriched TFs was assigned. In the table there are presented values of median correlations between TF coding gene expression and expression of DEGs (.xlsx file).

Supplementary Table S9. Transcription factor binding sites detected within six top 'glioma TADs*'.* With the use of BMO tool on eight glioma samples of various grades the TF binding sites were detected in the open chromatin regions marked with ATAC-seq.

TADs numbers	TFBS				
	E2F1	HMX1	PAX5	REST	ZBTB6
507	61	0	64	18	8
1101	16	0	30	0	20
1366	9	0	38	0	5
2007	146	0	137	43	51
2337	251	0	241	75	74
2343	0	1	2	0	0

Supplementary Table S10. DEGs with bivalent chromatin signals. DEGs simultaneously marked by H3K27me3 and H3K4me3 histone modifications at their promoters in GBM samples (.xlsx file).

Supplementary Table S11. Cohort-wide copy number events at a gene level. The samples are color-coded according to the grade of the glioma, and the rows contain genes. Gene amplifications are assigned a value of 1, gene deletions are assigned a value of -1, and neutral events are assigned a value of zero (.xlsx file).

Supplementary Table S12. Correlation between expression levels of the 117 genes contacting differentially acetylated enhancers and acetylation peak heights of those enhancers.

	Spearman correlation rho (p)	Median Spearman rho for permuted pairs
all grades together	0.45 (0.04), sd = 0.34	
GI (PA)	0.43 ($1.1e^{-6}$)	-0.01, sd = 0.09
GII/III (DA)	0.3 (0.001)	0, sd = 0.1
GIV (GBM/pGBM)	0.27 (0.003)	0.02, sd = 0.1

Supplementary Table S13. DEGs in contact with enhancers. Nine DEGs (PA vs GBM) having contacts with enhancers having both differential H3K27ac and methylation (DNA methylation) signals (between PA vs GBM). Table also shows the correlation levels between H3K27ac and DNA methylations of the enhancers contacting those DEGs (.xlsx file).

Supplementary Dataset

Supplementary Dataset S1. Genes dataset proteinatlas.tsv downloaded from proteinatlas.org (.txt Supplementary file).

References

1. Stępniak, K. *et al.* Mapping chromatin accessibility and active regulatory elements reveals pathological mechanisms in human gliomas. *Nat. Commun.* **12**, 3621 (2021).

2. Love, M. I., Huber, W. & Anders, S. Moderated estimation of fold change and dispersion for RNA-seq data with DESeq2. *Genome Biol.* **15**, 550 (2014).
3. Won, H. *et al.* Chromosome conformation elucidates regulatory relationships in developing human brain. *Nature* **538**, 523–527 (2016).
4. Stepniak, K. *et al.* Mapping chromatin accessibility and active regulatory elements reveals new pathological mechanisms in human gliomas. *bioRxiv* 867861 (2019).
5. Gaspar, J. M. Improved peak-calling with MACS2. *bioRxiv* 496521 (2018) doi:10.1101/496521.
6. Grant, C. E., Bailey, T. L. & Noble, W. S. FIMO: Scanning for occurrences of a given motif. *Bioinformatics* **27**, 1017–1018 (2011).
7. Johnston, M. J. *et al.* High-resolution structural genomics reveals new therapeutic vulnerabilities in glioblastoma. *Genome Res.* **29**, 1211–1222 (2019).
8. Subramanian, A. *et al.* Gene set enrichment analysis: A knowledge-based approach for interpreting genome-wide expression profiles. *Proc. Natl. Acad. Sci.* **102**, 15545 LP – 15550 (2005).
9. Huang, D. W., Sherman, B. T. & Lempicki, R. A. Systematic and integrative analysis of large gene lists using DAVID bioinformatics resources. *Nat. Protoc.* **4**, 44–57 (2009).
10. Chen, E. Y. *et al.* Enrichr: interactive and collaborative HTML5 gene list enrichment analysis tool. *BMC Bioinformatics* **14**, 128 (2013).
